# FAM110A promotes mitotic spindle formation by linking microtubules with actin cytoskeleton

**DOI:** 10.1073/pnas.2321647121

**Published:** 2024-07-12

**Authors:** Cecilia Aquino-Perez, Mahira Safaralizade, Roman Podhajecky, Hong Wang, Zdenek Lansky, Robert Grosse, Libor Macurek

**Affiliations:** ^a^Cancer Cell Biology, Institute of Molecular Genetics, Czech Academy of Sciences, Prague CZ14220, Czech Republic; ^b^Institute for Clinical and Experimental Pharmacology and Toxicology I, Medical Faculty, University of Freiburg, Freiburg 79104, Germany; ^c^Institute of Biotechnology, Czech Academy of Sciences, Biocev, Vestec CZ25250, Czech Republic; ^d^Centre for Integrative Biological Signaling Studies, University of Freiburg, Freiburg 79104, Germany

**Keywords:** mitosis, mitotic spindle, actin, microtubules, protein kinase

## Abstract

Precise segregation of chromosomes during mitosis requires assembly of a bipolar mitotic spindle followed by correct attachment of microtubules to the kinetochores. This highly spatiotemporally organized process is controlled by various mitotic kinases and molecular motors. We have recently shown that Casein Kinase 1 (CK1) promotes timely progression through mitosis by phosphorylating FAM110A leading to its enrichment at spindle poles. However, the mechanism by which FAM110A exerts its function in mitosis is unknown. Using structure prediction and a set of deletion mutants, we mapped here the interaction of the N- and C-terminal domains of FAM110A with actin and tubulin, respectively. Next, we found that the FAM110A-Δ40-61 mutant deficient in actin binding failed to rescue defects in chromosomal alignment caused by depletion of endogenous FAM110A. Depletion of FAM110A impaired assembly of F-actin in the proximity of spindle poles and was rescued by expression of the wild-type FAM110A, but not the FAM110A-Δ40-61 mutant. Purified FAM110A promoted binding of F-actin to microtubules as well as bundling of actin filaments in vitro. Finally, we found that the inhibition of CK1 impaired spindle actin formation and delayed progression through mitosis. We propose that CK1 and FAM110A promote timely progression through mitosis by mediating the interaction between spindle microtubules and filamentous actin to ensure proper mitotic spindle formation.

## Significance

Mitotic spindles are essential for correct segregation of genetic material during cell division. Whereas microtubules represent the main building blocks of mitotic spindles, presence of actin microfilaments in this structure has long been debated. Recent advances in visualization of actin dynamics in living cells enabled detection of transient actin structures during mitosis. Here, we show that a so far unexplored FAM110A protein has unique properties that allow it to bind actin and tubulin with the opposite ends and thus crosslink both cytoskeletal systems. FAM110A present at the poles promotes formation of mitotic spindles by mediating interaction between the actin filaments and microtubules. Biochemical properties are conserved in the FAM110 family and thus this study sets ground for future discoveries.

Precise distribution of genetic information during cell division relies on formation of the bipolar mitotic spindle that orchestrates segregation of the chromosomes into daughter cells. Mitotic spindles comprise kinetochore, polar, and astral microtubules that attach to the sister chromatids, segregate them toward the opposite poles and position them within the cells, respectively. Timing of these mitotic events is controlled by a spindle assembly checkpoint (SAC) that monitors the correct attachment of all kinetochores to spindle microtubules (1). Cortical actin microfilaments are important for correct positioning of the mitotic spindles and also for formation of the contractile actomyosin ring during cytokinesis (2, 3). Whereas the essential function of microtubules in formation of mitotic spindles is well established, the function of actin microfilaments in this process has long remained controversial and only recently begins to emerge together with improved techniques for F-actin detection (2, 4–6). Centrosomes represent major microtubule-organizing centers in cells but they can also promote assembly of actin filaments (7). The burst of actin polymerization around the spindle poles in early anaphase limits the nucleation of microtubules (8). This inverse regulation of F-actin and microtubule cytoskeleton during mitotic exit resembles the situation in activated B lymphocytes and in *Xenopus* egg extracts where branched actin filaments act as a physical barrier to MT elongation (9–11). In addition, recent advances in F-actin visualization allowed identification of transient actin structures in several subcellular compartments including the nucleus and mitotic spindle (12–16). Transient actin polymerization from centrosomes in early mitosis was shown to precede and guide the formation of kinetochore MTs (12). Interfering with the Arp2/3-dependent F-actin formation delayed chromosomal alignment at metaphase plate and impaired segregation of the sister chromatids (12). Finally, the perinuclear actin restrains the centrosome separation in prophase cells and limits chromosome scattering after nuclear envelope breakdown (NEB) (17–19).

We have recently shown that FAM110A localizes to the spindle poles and that its depletion delays progression through mitosis (20). In addition, mitotic function of FAM110A was dependent on its phosphorylation by CK1 that promoted its localization to the spindle poles in metaphase (20). Here, we report that mitotic function of FAM110A depends on its ability to bind actin and microtubules through its N- and C-terminal domains, respectively. Depletion of FAM110A impaired assembly of the spindle actin and delayed the transition to anaphase. This phenotype was rescued by the wild-type FAM110A but not by the FAM110A-Δ40-61 mutant deficient in actin binding. Similarly, inhibition of CK1 impaired spindle actin formation and impaired mitotic progression. Importantly, the defect in spindle actin assembly caused by inhibition of CK1 was rescued by FAM110A-S252E mutant, suggesting that the impact of CK1 on actin cytoskeleton in mitosis is mediated by FAM110A. We propose that FAM110A promotes mitotic spindle formation by providing the interaction between spindle actin and kinetochore microtubules.

## Results

### FAM110A Interacts with Actin and Tubulin through Its N- and C-Terminal Domains.

**Fig. 1.**
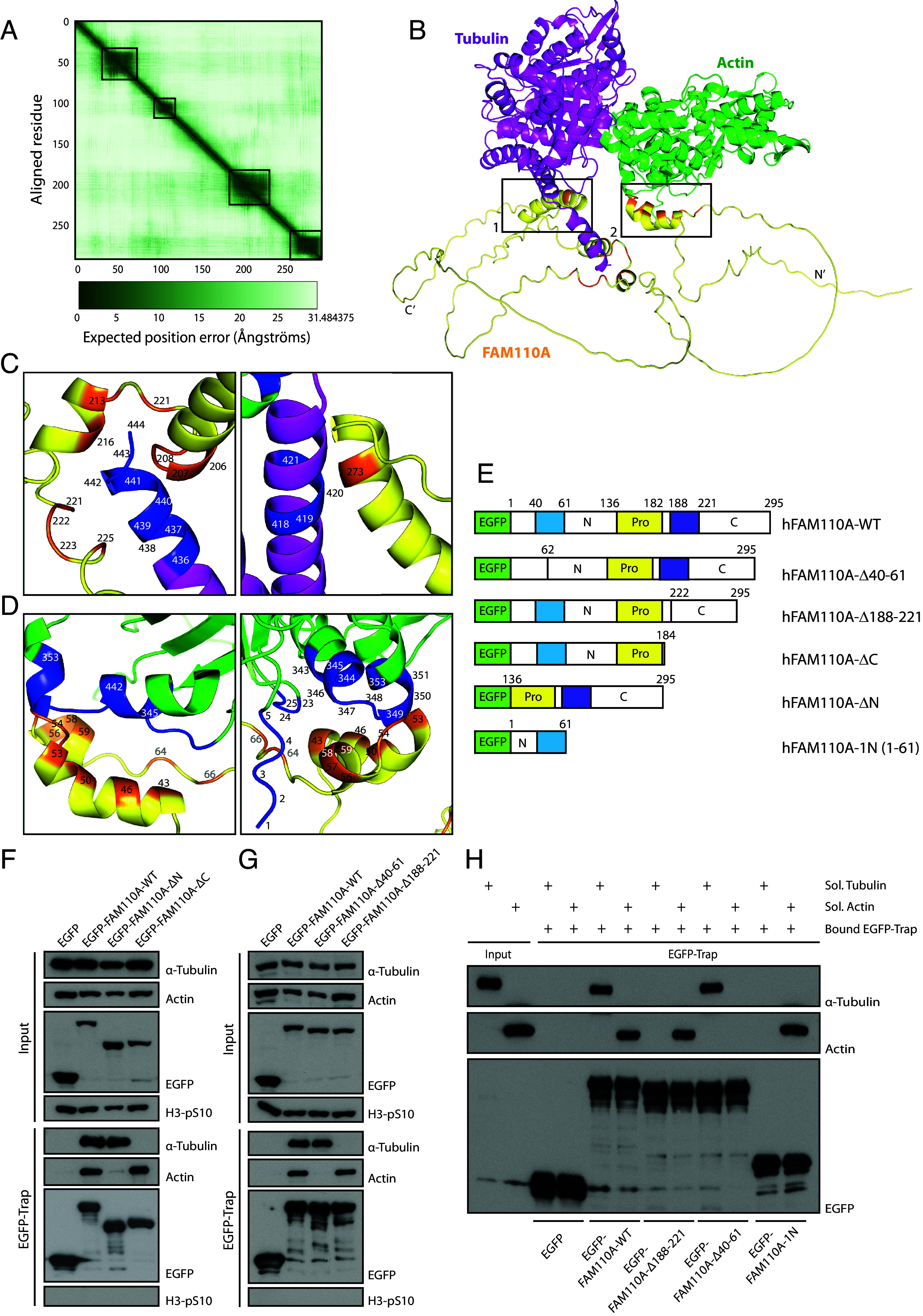
FAM110A interacts with actin and tubulin through its N- and C-terminal domains. (*A*) FAM110A predicted alignment error (PAE) for assessment of protein interdomain accuracy. (*B*) AlphaFold2 prediction model of FAM110A (yellow) interaction with actin (green) and tubulin (magenta). Predicted binding interface regions in the FAM110A model are shown in orange. Markup A1 shows that the predicted binding interface for tubulin (F206-A225), markup A2 for actin (V43-P66). (*C*) A close-up of the direct interaction between tubulin (magenta) and FAM110A (yellow). Predicted binding interaction interfaces in FAM110A are shown in orange while binding interfaces in tubulin are shown in blue. (*D*) A close-up of the direct interaction between actin (green) and FAM110A (yellow). Predicted binding interaction interface in FAM110A is shown in orange, while binding interface in actin is shown in blue. (*E*) Scheme of the EGFP‐FAM110A constructs used in the study. Numbering is based on human FAM110A. N, C, and Pro represent N‐terminal, C‐terminal, and Pro‐rich domains, respectively. The blue area represents conserved α-helix Ser41 to Glu-65, and the purple area represents conserved α-helix Ser-188 to Gly-221. (*F*) Immunoprecipitation assay performed in HEK293 cells transiently transfected with EGFP, EGFP-FAM110A-WT, and FAM110A variants −ΔN and −ΔC (*n* = 3). Antibody against pS10‐H3 was used as a marker of mitosis. (*G*) Immunoprecipitation assay performed in HEK293 cells transiently transfected with EGFP, EGFP-FAM110A-WT, and FAM110A variants -Δ40-61 and -Δ188-221 (*n* = 3). Antibody against pS10‐H3 was used as a marker of mitosis. (*H*) In vitro pull-down assay using GFP-trap beads coated with EGFP control, EGFP-FAM110A-WT, EGFP-FAM110A-Δ40-61, and EGFP-FAM110A-1N and purified soluble actin and tubulin monomers (*n* = 3).

In our previous work, we showed that FAM110A interacts with tubulin and actin during mitosis (20). Here, we aimed to map and functionally characterize the interaction of FAM110A with both cytoskeletal systems. First, we performed a sequence alignment that revealed a considerable level of conservation among the vertebrate orthologues of FAM110A showing the highest similarity in the N- and C-terminal regions separated by a more variable central region (*SI Appendix*, Fig. S1*A*). Analysis by AlphaFold2 software predicts that the Lys-39 to Asn-67, Leu-104 to Asp-112, Leu-191 to Arg-217, and Val-268 to Glu-289 regions of FAM110A fold into alpha-helix structures with low expected position error (Fig. 1*A*). Next, we used AlphaFold2 to predict possible direct protein–protein interactions of FAM110A with actin and tubulin (Fig. 1*B*). Interestingly, AlphaFold2 showed that interaction of tubulin could potentially be mediated by a conserved alpha-helix comprising residues 188–221 of FAM110A that are close to the previously reported phosphorylated sites promoting the binding of FAM110A to the spindle poles (20) (Fig. 1*C*). In addition, AlphaFold2 predicted interaction between actin and the alpha-helix comprising residues 41–65 of FAM110A (Fig. 1*D*). Based on these predictions we designed a panel of deletion mutants of EGFP-tagged FAM110A and performed immunoprecipitation assays to screen for their interaction partners in mitotic cells (Fig. 1*E*). In agreement with our previous observations, we confirmed that actin and tubulin bind to N- and C-terminal domains of FAM110A, respectively (Fig. 1*F*) (20). Moreover, we found that FAM110A-Δ188-221 failed to interact with tubulin, while it still bound actin (Fig. 1*G*). Next, we generated a series of truncations in the N-terminal domain of FAM110A and found that residues 40–61 were essential for actin binding (*SI Appendix*, Fig. S1 *B* and *C*). Indeed, we observed that FAM110A-Δ40-61 mutant was able to interact with tubulin, but not with actin (Fig. 1*G*). We also noted that the N- and C-terminal regions of FAM110A showed high sequence homology with its paralogues FAM110B and FAM110C (*SI Appendix*, Fig. S1*D*). Therefore, we mutated the corresponding regions in FAM110B and tested the impact on binding to actin (*SI Appendix*, Fig. S1*B*). We found that the wild-type FAM110B interacted with actin but the interaction was strongly reduced in FAM110B-Δ49-69 mutant (*SI Appendix*, Fig. S1*E*). Similarly, mutagenesis of the conserved F‐X‐X‐X‐F consensus docking motif in the FAM110B-FA mutant impaired the interaction with CK1 and with the tubulin, suggesting that FAM110A and FAM110B may share the general binding properties (*SI Appendix*, Fig. S1*E*) (20). We conclude that the N-terminal region of FAM110A represents an actin binding domain conserved in FAM110 family.

### Actin-Binding Domain Is Necessary for Mitotic Function of FAM110A.

**Fig. 2.**
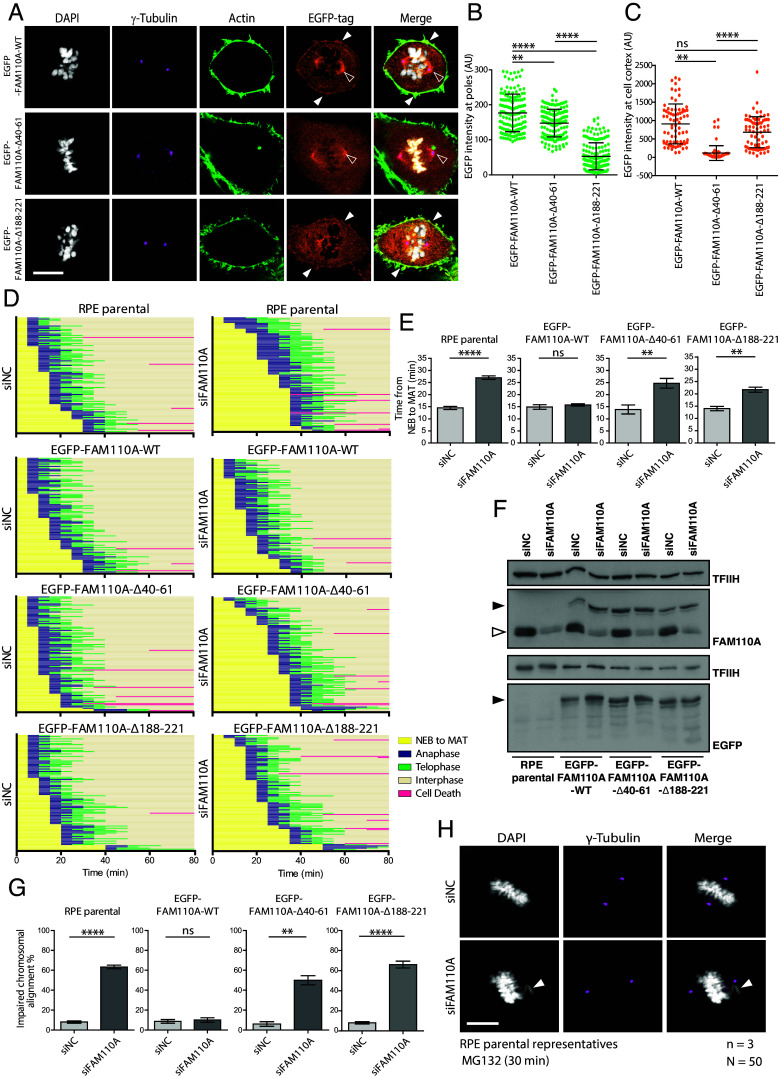
Actin-binding domain is necessary for mitotic function of FAM110A. (*A*) Cells stably expressing the EGFP-FAM110A-WT, EGFP-FAM110A-Δ40-61, and EGFP-FAM110A-Δ188-221 mutant were fixed and stained for DAPI, γ‐tubulin-647 and actin with phalloidin-568 and analyzed by confocal microscopy. Representative images of a single stack of metaphase cells are shown. Full arrowheads show colocalization with cell cortex actin, and the empty arrowhead shows enrichment at spindle poles. (Scale bar, 10 μm.) (*B*) Cells from *A* were used to quantify EGFP signal enrichment at spindle poles of metaphase cells using γ-tubulin-647 signal as a mask. Each dot represents one spindle pole (*N* = 50); shown is median ± SD (*n* = 3). Statistical significance was determined by one-way ANOVA (****P < 0.0001, **P < 0.01). (*C*) Cells from *A* were used to quantify EGFP signal enrichment at the cell cortex of metaphase cells using phalloidin-568 signal as a mask. Each dot represents one cell (*N* = 50); shown is median ± SD (*n* = 3). Statistical significance was determined by one-way ANOVA (****P < 0.0001, **P < 0.01). (*D*) RPE parental cells and stable cell lines expressing EGFP-FAM110A-WT, -Δ40-61 or -Δ188-221 mutants were transfected with indicated siRNAs and after 48 h, they were filmed in 5 min intervals. Time frame closest to the NEB was set as 0 min. Progression through mitosis was categorized as follows: NEB to metaphase‐to‐anaphase transition (MAT), anaphase, telophase, and interphase. Data from one of three experiments are shown, and each bar indicates one cell (*n* = 100). Cells that died during imaging are shown in pink. (*E*) Time from the NEB to MAT was quantified in cells from *D*. Shown is median ± SD (*n* = 3). Statistical significance was determined by one-way ANOVA (****P < 0.0001, **P < 0.01). (*F*) Representative immunoblot analysis of samples from *D* and *H*. The full arrow indicates migration of the wild type or mutants EGFP‐FAM110A and the empty arrow indicates endogenous FAM110A. (*G*) RPE parental cells or cells stably expressing the EGFP-FAM110A-WT, -Δ40-61, or -Δ188-221 mutants were transfected with control (siNC) or FAM110A siRNA and grown for 48 h. Cells were treated with MG132 for 30 min prior fixation. Impaired chromosomal alignment was scored in 50 metaphase cells per condition. Error bars indicate median ± SD. Statistical significance was determined by one-way ANOVA (*n* = 3, ****P < 0.0001 and **P < 0.01). (*H*) Representative RPE parental cells from H. Cells transfected with control or FAM110A siRNA were fixed with 4% PFA after 48 h, and stained for γ‐tubulin-647 and DAPI. White arrowheads show a misaligned chromosome. Metaphase cells were imaged by confocal microscopy. (Scale bar, 10 μm.)

Next, we aimed to determine whether the actin-binding domain is involved in mitotic function of FAM110A. To this end, we established RPE cells stably expressing the wild-type EGFP-FAM110A, the actin-binding deficient mutant EGFP-FAM110A-Δ40-61 and the tubulin-binding deficient mutant EGFP-FAM110A-Δ188-221. We found that deletion of the actin-binding domain reduced the localization of EGFP-FAM110-Δ40-61 in the cell cortex, but that its localization at spindle poles during mitosis was preserved (Fig. 2 *A*–*C* and *SI Appendix*, Fig. S2 *B* and *C*). In contrast, we observed that deletion of the tubulin-binding domain significantly reduced the level of EGFP-FAM110A-Δ188-221 mutant at the spindle poles but it did not affect its localization at the cell cortex (Fig. 2 *A*–*C*). This observation is consistent with our previous report that localization of FAM110A to the spindle poles is mediated by its C-terminal domain (20). By measuring a fraction of cells positive for phosphorylated mitotic marker MPM2 using flow cytometry, we determined that EGFP-FAM110A-Δ40-61 and EGFP-FAM110A-Δ188-221 mutants failed to rescue the mitotic delay caused by depletion of endogenous FAM110A (*SI Appendix*, Fig. S2*A*). Similarly, live microscopy revealed that the wild-type FAM110A but not EGFP-FAM110A-Δ40-61 nor the EGFP-FAM110A-Δ188-221 mutant rescued the progression from the NEB to metaphase-to-anaphase transition (NEB to MAT) in cells with depleted FAM110A (Fig. 2 *D*–*F*). To address the consequences of the mitotic delay, we treated FAM110A-depleted cells briefly with the proteasome inhibitor MG132 and scored the chromosomal alignment in metaphase cells. In agreement with our previous report, about 60 % of the FAM110A-depleted cells showed chromosomal misalignments in metaphase (Fig. 2 *G* and *H*) (20). Importantly, the ectopic FAM110A-WT fully rescued the chromosomal alignment in metaphase cells, while the EGFP-FAM110A-Δ40-61 and EGFP-FAM110A-Δ188-221 mutants failed to do so (Fig. 2*G*). Further, we noted that depletion of FAM110A reduced the pole-to-pole distance and caused spindle pole fragmentation suggestive of dysregulation of the mechanical forces acting at the spindle in metaphase cells (*SI Appendix*, Fig. S2 *D*–*G*) (21). Depletion of FAM110A also slightly reduced microtubule density of the metaphase spindles (*SI Appendix*, Fig. S2 *H* and *I*). Importantly, EGFP-FAM110A-Δ40-61 as well as EGFP-FAM110A-Δ188-221 mutants failed to rescue the spindle size and the integrity of the spindle poles (*SI Appendix*, Fig. S2 *D*–*I*). Combined, these data suggest that FAM110A and its interaction with actin and tubulin are needed early in mitosis prior to satisfaction of the SAC.

### FAM110A Promotes Interaction between Kinetochore MTs and Spindle Actin.

**Fig. 3.**
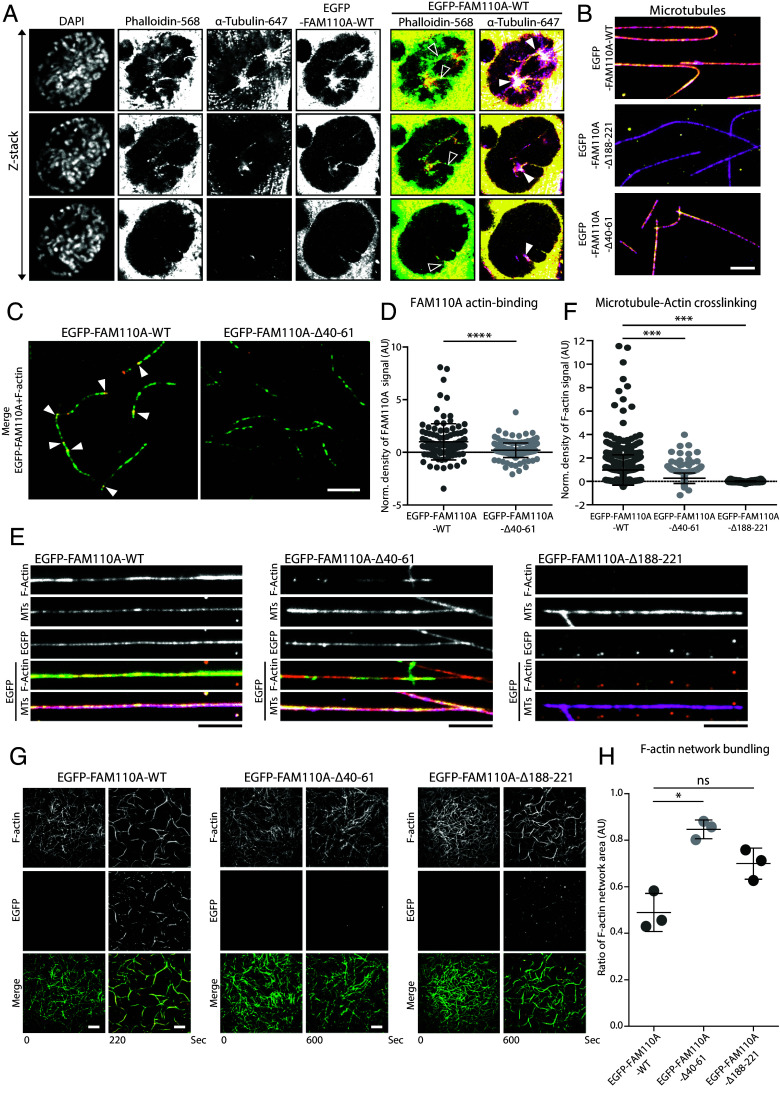
FAM110A promotes interaction between kinetochore fibers and spindle actin. (*A*) Asynchronously growing RPE cells stably expressing EGFP-FAM110A-WT were fixed and probed with Alexa-568-labeled phalloidin and α-tubulin-Alexa-647. Shown are representative images of prophase cells. Individual z‐stacks were taken every 0.5 µm and processed with Airyscan. EGFP-FAM110A-WT (orange) colocalization with spindle actin (green) is marked by empty arrowheads, while EGFP-FAM110A-WT (yellow) colocalization with spindle microtubules (magenta) is marked with white arrowheads. (Scale bar, 10 μm.) (*B*) Representative images of in vitro assay showing binding of the purified EGFP-FAM110A-WT, EGFP-FAM110A-Δ40-61, and EGFP-FAM110A-Δ188-221 (yellow for all variants) to immobilized MTs (magenta). (Scale bar, 5 μm.) (*C*) In vitro assay showing purified mitotic EGFP-FAM110A-WT and EGFP-FAM110A-Δ40-61 (orange) binding to F-actin (green). Biotinylated F-actin stabilized with fluorescently labeled phalloidin was attached to the coverslip surface via anti-biotin antibodies. FAM110A variants (400 nM) were flushed into the channel and imaged for 10 min; shown is the last frame of the assay. (Scale bars, 10 μm.) (*D*) Quantification of *C*. Plotted is the normalized density of EGFP signal on the F-actin 10 min after addition of EGFP-FAM110A variants. Error bars indicate median ± SD. Statistical significance was determined by the *t* test (*n* = 286 actin filaments in three independent experiments, ****P < 0.0001). (*E*) In vitro assay for MT-Actin crosslinking. MTs were polymerized and immobilized on the coverslip and incubated with purified EGFP-FAM110A-WT, -Δ40-61, or -Δ188-221. Subsequently, F-actin was flushed in the channel and crosslinking was imaged after 10 min. EGFP-FAM110A variants were shown in orange with F-actin (green) and MTs (magenta). (Scale bars, 5 μm.) (*F*) Quantification of *E*. Plotted is the normalized density of the F-actin signal on the MTs after 10 min of F-actin addition. Error bars indicate median ± SD. Statistical significance was determined by the *t* test (*n* = 1,207 microtubules in three experiments, ****P < 0.0001). (*G*) Representative images of F-actin bundling in vitro assay showing F-actin (green) bundling upon the addition of purified mitotic EGFP-FAM110A-WT, -Δ40-61 or -Δ188-221 (orange). (Scale bar, 10 μm.) (*H*) Quantification of *G*. Plotted is the last time point of the normalized F-actin network area after addition of respective EGFP-FAM110A variants. Error bars indicate median ± SD. Statistical significance was determined by the one-sample *t* test (*n* = 3, *P < 0.05).

As depletion of FAM110A or deletion of its actin-binding domain slowed down the progression through early mitosis and impaired chromosomal alignment, we hypothesized that FAM110A may be involved in formation of the recently described spindle actin structures and their interaction with the spindle microtubules (12). To test this, we imaged fixed RPE cells stably expressing the wild-type EGFP-FAM110 using confocal microscopy. We found that the EGFP-FAM110A colocalizes with F-actin as well as with the spindle microtubules in the prophase cells (Fig. 3*A*).

Motivated by the immunoprecipitation results and by the localization pattern of the mitotic FAM110A, we aimed to assay the interaction of FAM110A in the context of actin and tubulin polymers. To this end, we purified the wild-type EGFP-FAM110A-WT and the EGFP-FAM110A-Δ40-61 and EGFP-FAM110A-Δ188-221 mutants from mitotic cells and performed several in vitro binding assays. First, we noted that the wild-type EGFP-FAM110A and EGFP-FAM110A-Δ40-61 mutant were able to bind to microtubules, whereas the interaction was completely lost in EGFP-FAM110A-Δ188-221 mutant (Fig. 3*B*). In addition, we observed that the wild-type EGFP-FAM110A can interact with F-actin and this interaction was severely reduced in EGFP-FAM110A-Δ40-61 mutant (Fig. 3 *C* and *D*). Next, we found that the wild-type EGFP-FAM110A efficiently promoted crosslinking of F-actin to the immobilized microtubules (Fig. 3 *E* and *F*). In contrast, this interaction of F-actin with microtubules was severely reduced in the presence of EGFP-FAM110A-Δ40-61 and EGFP-FAM110A-Δ188-221 mutants (Fig. 3 *E* and *F*). To assess the effect of FAM110A on actin–actin bundling, we flushed the F-actin filaments into the measurement channel followed by purified EGFP-FAM110A variants and then imaged for a total of 10 min (Fig. 3 *G* and *H*). We observed that the wild-type EGFP-FAM110A reduced the ratio of the F-actin network area, indicating that it promoted bundling of the F-actin filaments, whereas the F-actin network area was not significantly reduced in the presence of EGFP-FAM110A-Δ40-61 mutant (Fig. 3*H*). Finally, we noted that EGFP-FAM110A-Δ188-221 mutant was able to significantly promote F-actin bundling (Fig. 3 *G* and *H* and Movie S1). We conclude that FAM110A can mediate interactions between microfilaments as well as between F-actin and microtubules both in vitro and in cells.

### FAM110A Promotes Spindle Actin Formation.

**Fig. 4.**
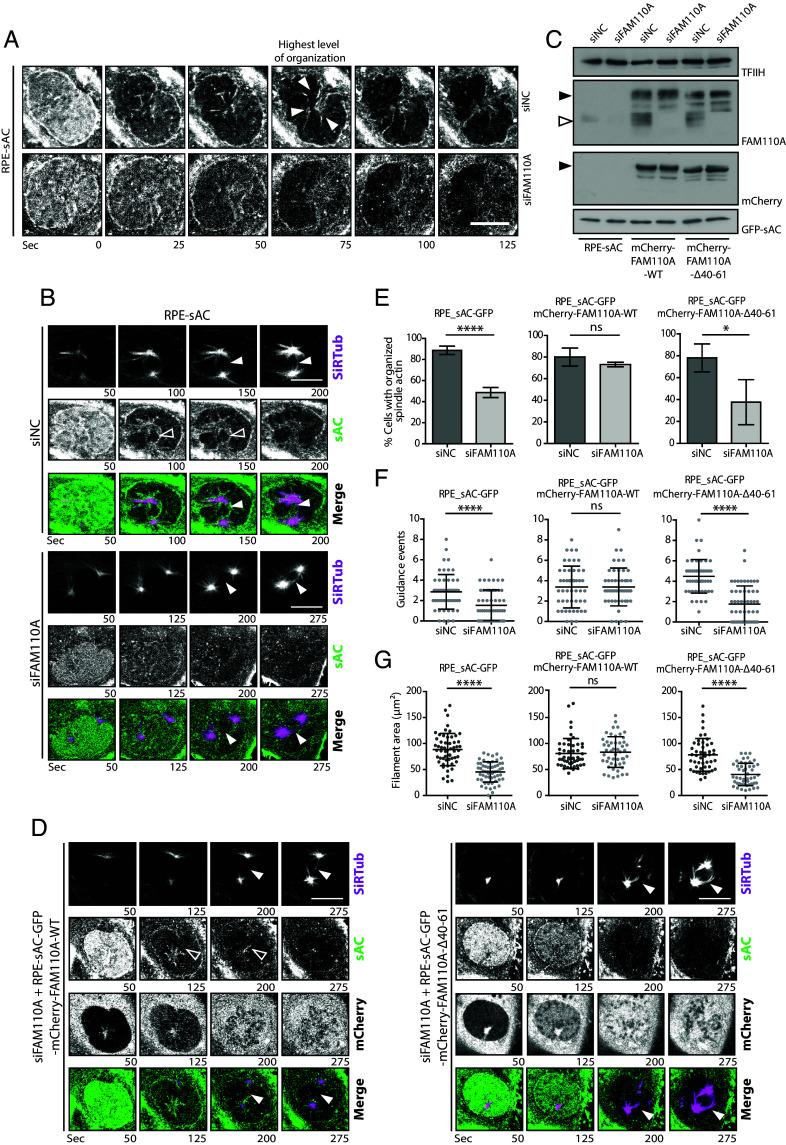
FAM110A promotes formation and organization of the spindle actin. (*A*) Representative live image sequences of RPE-sAC cells transfected with control (siNC) or FAM110A siRNA. Shown is GFP channel and numbers show time in seconds, time 0 was set to indicate NEB at the onset of prophase. Arrowheads indicate spindle actin with the highest level of organization. (Scale bar, 10 μm.) (*B*) Representative live image sequences of RPE-sAC cells transfected with control (siNC) or FAM110A siRNA and MT stained by SiR-Tub. The arrowhead shows a kinetochore MT fiber (magenta), the empty arrowhead shows event where spindle actin (green) preceded formation of the kinetochore MT. (Scale bars, 10 μm.) (*C*) Immunoblot analysis of parental RPE-sAC, RPE-sAC-mCherry‐FAM110A‐WT or RPE-sAC-mCherry-FAM110A-Δ40-61 cells transfected with control (siNC) or FAM110A siRNA and incubated with doxycycline. The empty arrow indicates endogenous FAM110A; the full arrow shows mCherry‐FAM110A. (*D*) Representative live image sequences of RPE-sAC-mCherry-FAM110A-WT and RPE-sAC-mCherry-FAM110A-Δ40-61 cells transfected with FAM110A siRNA. The arrowhead shows a kinetochore MT fiber; the empty arrowhead shows event where spindle actin preceded formation of kinetochore MT. (Scale bars, 10 μm.) (*E*) Quantification of spindle actin formation and organization in *B* and *D*. Plotted is the percentage of cells that presented an organized actin spindle for each independent repetition ± SD. Statistical significance was determined by the *t* test (*n* = 3, *N* = 25, ****P < 0.0001, *P < 0.05). (*F*) Quantification of actin-MT guidance events in *B* and *D*. Plotted is the number of events when spindle actin preceded growth of a kinetochore MT per cell. Bars indicate mean ± SD, each dot represents a single cell. Statistical significance was determined by the *t* test (*n* = 3, *N* = 25, ****P < 0.0001). (*G*) Quantification of the spindle actin area in *B* and *D* using Imaris software. Plotted is the total area covered by the organized spindle actin structure (µm^2^). Bars indicate mean ± SD, each dot represents a single cell. Statistical significance was determined by the *t* test (*n* = 3, *N* = 25, ****P < 0.0001).

Whereas spindle microtubules represent a prominent structure clearly detectable throughout the mitosis, formation of the mitotic F-actin structures is transient (8, 12, 17). To visualize formation of the spindle actin and its interaction with the spindle microtubules, we used RPE-sAC-GFP stably expressing a shuttling-actin-chromobody, labeled microtubules with SiR-Tubulin and performed live cell imaging (13). Consistent with a previous report, we observed that F-actin formed rapidly after NEB and frequently preceded growth of the spindle microtubules (Fig. 4 *A*, *B*, *E*, and *F* and Movies S1 and S2) (12). To visualize the impact of FAM110A on dynamics of the spindle actin, we depleted FAM110A in RPE-sAC-GFP cells and followed them by live imaging microscopy during the mitotic progression. We found that the percentage of cells with clearly formed spindle actin structure was decreased upon depletion of endogenous FAM110A (Fig. 4 *A*, *B*, *E*, and *F* and Movie S3). Next, we stably transfected RPE-sAC-GFP cells with plasmids allowing for a doxycycline-inducible mCherry-FAM110A-WT or mCherry-FAM110A-Δ40-61. First, we used confocal microscopy and verified that the localization of the mCherry-tagged FAM110A variants in the stable cell lines was similar as the EGFP-tagged counterparts (*SI Appendix*, Fig. S3*A*). Next, we found that the fraction of cells with organized spindle actin was fully rescued by induction of the wild-type mCherry-FAM110A expression in cells transfected with FAM110A siRNA, while spindle actin formation was impaired in cells expressing mCherry-FAM110-Δ40-61 mutant (Fig. 4 *C*–*E* and Movies S4 and S5). To independently confirm these observations obtained by visual inspection of the live cell imaging data, we analyzed the dataset using Imaris software. This analysis revealed that FAM110A depletion reduced the spindle actin area as well as the total F-actin filament length in cells (Fig. 4*G* and *SI Appendix*, Fig. S4*B*). Similarly, we found that the number of branch points in F-actin was strongly reduced upon depletion of FAM110A (*SI Appendix*, Fig. S4*C*). Importantly, expression of the wild-type mCherry-FAM110A but not mCherry-FAM110-Δ40-61 mutant rescued the total spindle actin area, F-actin length, and branching (Fig. 4*G* and *SI Appendix*, Fig. S4 *B* and *C*). Finally, we manually scored for the guidance events where actin filament preceded the formation of microtubules and we observed a significantly reduced number of the guidance events per cell upon depletion of FAM110A (Fig. 4*F*). Similarly as the other phenotypes, MT guidance by actin was rescued by the wild-type mCherry-FAM110 but not mCherry-FAM110-Δ40-61 mutant (Fig. 4*F*). Alongside we also depleted the Cytoplasmic Linker associated protein 2 (CLASP2) that has previously been implicated in cross-linking F-actin and microtubules (22, 23), and analyzed the effects in the organization of spindle actin. In contrast, we observed that depletion of CLASP2 did not significantly affect any of the parameters described above, suggesting that the spindle actin formation may specifically depend on the cross-linking activity of FAM110A (*SI Appendix*, Fig S3 *B*–*H* and Movie S6). We conclude that the overall complexity of the spindle actin organization is promoted by FAM110A as well as its ability to mediate the interaction between actin filaments and microtubules.

### Casein Kinase 1 Regulates Spindle Actin through Modification of FAM110A.

**Fig. 5.**
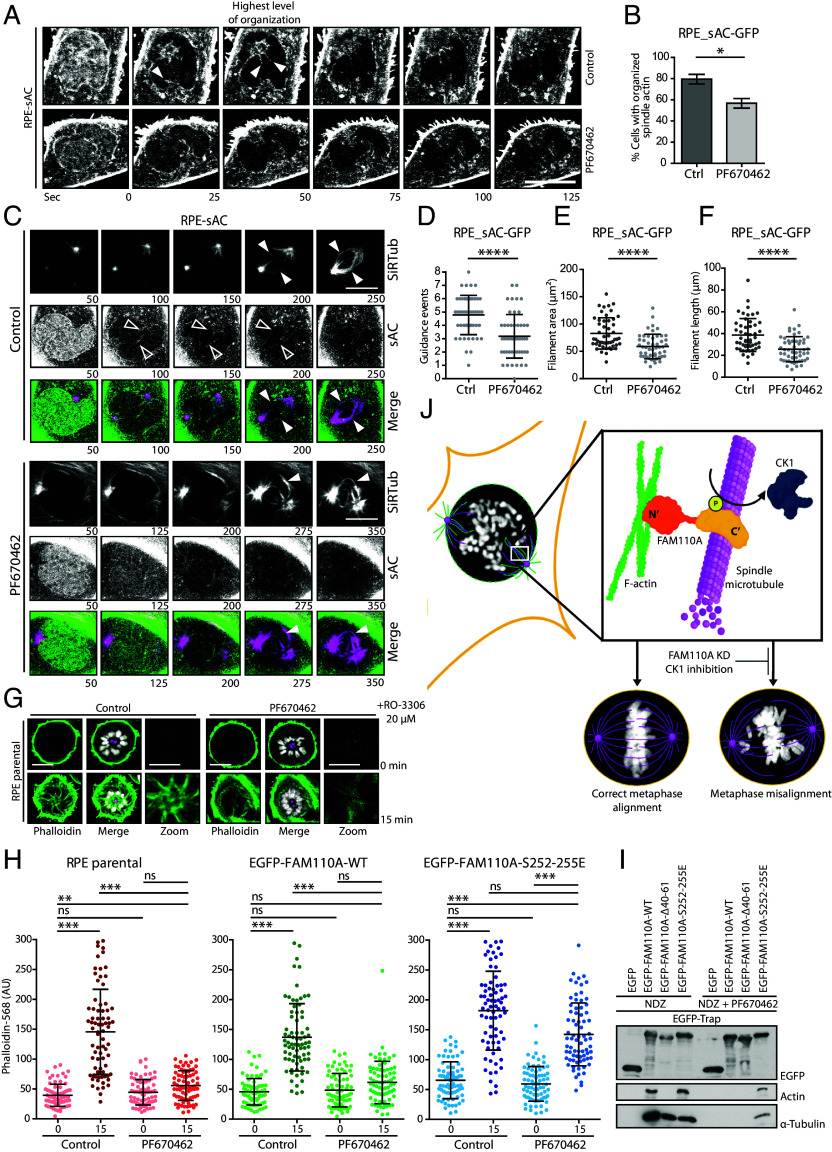
CK1 regulates spindle actin by promoting FAM110A interaction with spindle microtubules. (*A*) Representative live image sequence of RPE-sAC-GFP cells treated with DMSO or with CK1 inhibitor PF670462 (1 µM). Cells were imaged every 25 s, time 0 was set to indicate NEB. (Scale bar, 10 μm.) (*B*) Quantification of *A*. Plotted is percentage of cells that presented an organized actin spindle for each independent repetition ± SD. Statistical significance was determined by the *t* test (*n* = 3, *N* = 25, *P < 0.05). (*C*) Representative live image sequences of RPE-sAC-GFP cells treated with DMSO or PF670462 (1 µM) and labeled with SiR-Tub. The arrowhead shows a kinetochore MT fiber; the empty arrowhead shows event where spindle actin preceded formation of kinetochore MT. (Scale bars, 10 μm.) (*D*) Quantification of the actin-MT guidance events in *C*. Plotted is the number of events when spindle actin preceded growth of a kinetochore MT per cell. Bars indicate mean ± SD, each dot represents a single cell. Statistical significance was determined by the *t* test (*n* = 3, *N* = 25, ****P < 0.0001). (*E*) Quantification of the spindle actin filament area in *C*. Plotted is the total sum of the area covered by the organized F-actin (µm^2^). Each dot represents a single cell, bars show mean ± SD observed per cell. Statistical significance was determined by the *t* test (*n* = 3, *N* = 25, ****P < 0.0001). (*F*) Quantification of the spindle actin length in *C*. Plotted is the total sum of the length of all detected filaments per cell (µm). Each dot represents a single cell, bars indicate mean ± SD. Statistical significance was determined by the *t* test (*n* = 3, *N* = 25, ****P < 0.0001). (*G*) Representative images of F-actin formation during the mitotic exit. RPE cells were arrested in prometaphase using STLC (5 µM) and were treated with DMSO or PF6700462 (1 µM). Then cells were forced to exit mitosis by RO-3306 and fixed after 15 min. DNA was stained with DAPI (white), actin with phalloidin (green), and centrosomes were labeled with γ-tubulin (magenta). Scale bars indicate 10 μm for full panels and 5 μm for zoom panels. (*H*) Quantification of actin growth around the centrosomes after forced mitotic exit from (*G* and *H*). Plotted is mean of Phalloidin-568 intensity around the monopolar centrosome at 0 or 15 min time point ± SD (each dot represents the mean value of all stacks of each single cell). Statistical significance was determined by ANOVA (*n* = 3) (*N* = 25) (***P < 0.001, **P < 0.01). (*I*) HEK293 cells transfected with indicated constructs were arrested in mitosis with NDZ and treated or not with PF670462 for 6 h. Pull-down from cell extracts was performed using GFP-Trap, and binding of tubulin and actin was determined by immunoblotting (*n* = 2). (*J*) Schematic representation showing FAM110A function in crosslinking the spindle actin and spindle microtubules in early mitosis. FAM110A is phosphorylated by CK1, which promotes its enrichment at spindle poles. Subsequently, FAM110A mediates spindle actin formation by F-actin bundling and mitotic spindle formation by crosslinking of MTs with F-actin. Depletion of FAM110A or inhibition of CK1 delays progression through mitosis and impairs chromosomal in metaphase.

We have previously shown that mitotic function of FAM110A is regulated by phosphorylation of its C-terminal region by CK1, and therefore, we aimed to test whether CK1 activity regulates the spindle actin formation (20). To this end, we filmed RPE-sAC-GFP cells in the presence of DMSO or PF670462 and found that inhibition of CK1 impaired organization of the spindle actin in prophase cells (Fig. 5 *A* and *B* and Movie S7) (24) and significantly reduced the number of the actin-MT guidance events (Fig. 5 *C* and *D* and Movies S8 and S9). Imaris analysis further confirmed that the F-actin area around the spindle poles, the filament length, and the number of F-actin guidance events were reduced upon inhibition of CK1 (Fig. 5 *E* and *F*). Similarly, we observed that inhibition of CK1 or siRNA-mediated depletion of CSNK1D reduced F-actin assembly during the mitotic exit forced by inhibition of CDK1 (Fig. 5*G* and *SI Appendix*, Fig. S4*D*) (8). Next, we employed this assay to determine the contribution of FAM110A to mediate the impact of CK1 on mitotic actin assembly. We used the RPE cells stably expressing the wild-type EGFP-FAM110A or EGFP-FAM110A-S252-255E mutant that has previously been reported to rescue the mitotic arrest caused by inhibition of CK1 (20). Interestingly, we found that expression of EGFP-FAM110A-S252E mutant (but not of the wild-type EGFP-FAM110A) rescued the defect in actin polymerization upon inhibition of CK1 (Fig. 5*H* and *SI Appendix*, Fig. S4*E*). Finally, we immunoprecipitated FAM110A variants from the mitotic cells treated with the PF670462 inhibitor and found that the interaction of EGFP-FAM110A-WT with actin and tubulin was strongly reduced whereas it was preserved in the phosphomimicking EGFP-FAM110A-S252-255E mutant (Fig. 5*I*). We conclude that FAM110A phosphorylated by CK1 mediates the interaction between spindle actin and mitotic spindle and thus promotes timely progression through mitosis.

## Discussion

Potential involvement of F-actin in spindle assembly and function represents a longstanding question in the field (2, 6). Whereas F-actin was described at acentrosomal meiotic spindles in frog and mouse oocytes (25–28), its presence at mitotic spindles in somatic animal cells has long remained controversial. Electron microscopy revealed a presence of F-actin in mitotic spindles (29–31), but these observations were not confirmed by phalloidin staining or immunofluorescence microscopy (32, 33). This discrepancy might be explained by several factors, including a high background signal of cytoplasmic F-actin and by the cortical actin that can mask detection of highly dynamic structures at mitotic spindles. Development of a fluorescent GFP-Utr-CH probe for F-actin detection based on the actin-binding domain of utrophin revealed formation of the spindle actin cables in epithelial cells in *Xenopus* (34, 35). Similarly, LifeAct probe showed F-actin nucleation from the interphase centrosomes as well as from the spindle poles during the anaphase (7, 8). More recently, a fluorescently labeled actin nanobody allowed F-actin visualization at mitotic spindle (12, 13, 36). This sensitive tool revealed that transient F-actin structures around the spindle poles are formed already in early mitosis and are followed by a more prominent actin polymerization during mitotic exit (12). In this study, we investigated mechanisms contributing to the spindle actin formation and function. We have found that spindle actin assembly is impaired upon depletion of FAM110A, a protein we previously identified at mitotic spindle poles (20). Proteomic analysis, immunoprecipitation assays, and domain mapping revealed that FAM110A binds actin and tubulin by its N- and C-terminal domains, respectively. Importantly, deletion of the actin-binding region of FAM110A suppressed its impact on the spindle actin assembly, impaired chromosomal alignment and extended the duration of mitosis. High-resolution confocal microscopy of RPE cells expressing the actin nanobody and treated with Sir-Tubulin revealed frequent events of close association between the spindle actin and spindle microtubules; and that usually F-actin preceded growth of the microtubule. This observation is consistent with the previously proposed model of guidance of the kinetochore microtubules by F-actin during prophase (12). In agreement with previous reports we also observed a complex actin network around the spindle poles during exit from mitosis (8). A dense criss-cross actin meshwork and branched actin filaments can block microtubule growth in several conditions including the anaphase (8, 9, 11). Our live cell imaging revealed that spindle actin formed concomitantly with NEB and was highly dynamic and disassembled in approximately 150 s. This suggests that the transient spindle actin structure may provide the initial guidance clues for individual kinetochore MTs but later may disassemble to provide space for massive MT growth during metaphase. A second wave of the branched actin assembly in anaphase corresponds to the reduced density of MTs during mitotic exit. Interestingly, we noticed that the spindle F-actin formation during the mitotic exit was reduced upon depletion of FAM110A suggesting that FAM110A may regulate actin cytoskeleton throughout mitosis.

To address how FAM110A regulates the microtubules and actin cytoskeleton, we assayed the binding of the purified FAM110A in vitro. We observed that FAM110A mediated the interaction between microtubules and F-actin and also promoted bundling of the actin filaments. Both these activities were dependent on the actin-binding region of FAM110A. Moreover, we noticed that the EGFP-FAM110A pulled down FAM110A-FLAG, and therefore it is plausible that FAM110A dimers or oligomers could mediate the interaction between individual actin filaments. Nevertheless, the precise molecular basis of the FAM110A-mediated actin bundling remains to be addressed.

Finally, we investigated the pathways regulating the spindle actin formation. As we previously reported, FAM110A strongly interacts with and is phosphorylated by CK1 isoforms CSNK1D and CSNK1E; and therefore, we hypothesized that CK1 may influence the spindle actin formation (20). Indeed, we found that inhibition of CK1 activity with a small molecule compound impaired spindle actin assembly in prophase and during mitotic exit. Importantly, F-actin assembly in cells treated with CK1 inhibitor was rescued by expression of a phosphomimicking mutant EGFP-FAM110A-S252E but not by a wild-type FAM110A. These data confirm that CK1 controls mitotic progression by targeting FAM110A. Which of the two CK1 isoforms is involved in control of the mitotic functions of FAM110A remains currently unclear. We noticed that the combined depletion of CSNK1E and CSNK1D showed more pronounced mitotic phenotypes suggesting a partial redundancy of both CK1 isoforms. In summary, we propose a model in which CK1 activity promotes FAM110A localization at spindle poles leading to formation of spindle actin and its interaction with the spindle microtubules and allowing the timely progression through mitosis (Fig. 5*J*). In this study, we observed that the wild-type EGFP-FAM110A is strongly enriched at spindle poles and spindle actin but it localizes also in the cell cortex. A deletion mutant lacking the 40–61 region of FAM110A failed to rescue formation of the spindle actin in FAM110A-depleted cells. Most likely explanation of this observation is that FAM110A localized at spindle poles promotes spindle actin assembly by mediating the interaction between F-actin and MTs. In addition, EGFP-FAM110A-Δ188-221 mutant that normally localizes also in the cell cortex and is capable of F-actin bundling in vitro, failed to rescue the mitotic phenotypes caused by depletion of FAM110A suggesting that mitotic FAM110A acts independently of the actin dynamics at cell periphery. Finally, we used stable MT and F-actin polymers in the in vitro assays, and therefore, we could not determine potential impact of FAM110A and its associated proteins on the growth of the microtubules and microfilaments. These open questions remain to be addressed by future research.

## Materials and Methods

For additional information, please see *SI Appendix*, *SI Materials and Methods*.

### Cells.

Human hTERT-immortalized RPE1 cells (referred to as RPE1) were obtained from ATCC and were grown in DMEM supplemented with 6 % FBS, penicillin, and streptomycin in 5 % CO_2_ at 37 °C. RPE cells stably expressing EGFP-FAM110A or its mutants were generated by transfection of linearized plasmids followed by selection with geneticin for 3 wk and FACS of GFP-positive cells. RPE cells stably expressing the shuttling Actin-Chromobody (referred to as RPE-sAC) were generated using lentiviral transduction of the plasmid sAC-TagGFP2 and were described previously (13, 37). For doxycycline-inducible expression of FAM110A and its mutant, RPE-sAC cells were cotransfected with pSBtet-Pur-FAM110A or pSBtet-Pur-FAM110A-Δ40-61 and SP1-SB100X (Addgene ID: 154887) and selected by puromycin for 3 wk. All cells were regularly tested for mycoplasma infection using the MycoAlert kit (Lonza). Transfection of plasmid DNA was performed using Lipofectamine 2000 (Thermo Scientific) or by polyethylenimine. Transfection of Silencer Select siRNA (5 nM) oligonucleotides was performed using RNAiMAX (Thermo Scientific). Targeting sequence of the human FAM110A is CAAUACAAGGUUUUUGACA and has been validated previously (20).

### Prediction of Protein Structures by AlphaFold.

The interaction of FAM110A sequence with actin and tubulin was predicted from LocalColabFold with the alphafold2_multimer_v3 model. The interaction surface was identified by the presence of hydrogen bonds within 3.5 Å distance. The structure of FAM110A was predicted with AlphaFold 2–based software LocalColabFold with the default settings and amber relaxation. The structure was colored based on per-residues pLDDT scores. The higher the score, the more confident is the structure. Prediction models editing was performed using PyMol 2 software.

### Time-Lapse Microscopy and Data Analysis.

Parental RPE or RPE cells stably expressing EGFP-FAM110A-WT, -Δ40-61, or -Δ188-221 variants were transfected with control or specific siRNA oligonucleotides and seeded into Lab-TekII cover-glass chambers (Thermo Scientific). After 32 h posttransfection, cells were imaged every 5 min for up to 24 h using the Leica DMI6000 microscope equipped with N PLAN 40x/0.55 CORR DRY objective and with an 37 °C, 5% CO_2_ environmental chamber. Films were analyzed using LAS AF Lite software (Leica). Division kinetics were determined manually by counting each 5-min interval from the NEB to metaphase-to-anaphase transition. In total, 100 individual cells were quantified per condition in three independent experiments. Alternatively, RPE-sAC cells and RPE-sAC_mCherry-FAM110A-WT/Δ40-61 Dox-inducible stable cell lines were transfected with control or specific siRNA and incubated for 24 h. Then, transfected cells were split for protein collection and to seed in glass bottom dishes (Ibidi µ-Dish 35 mm -1.5H Glass Bottom Dish). After 32 h posttransfection, SirR-Tubulin (40 nM, Spirochrome) was added to the media and incubated for 1 h. Cells were monitored manually in search for cells with duplicated centrosomes with polarity right before NEB took place. The imaging plane where the nucleus has the biggest area was selected for single-slice imaging using definite focus mode. Cells were imaged every 25 s starting from 1 min before NEB until Metaphase plate was properly formed. All in vivo imaging was generated using a confocal laser scanning microscope (LSM800, Zeiss) equipped with an Airyscan detector using an oil 63X 1.4 NA objective. All RPE-sAC images were first processed by Zeiss Airyscan and then further analyzed and prepared using IMARIS Cell imaging software. Spindle actin morphology and dynamics were analyzed using the IMARIS 10.1.0 filament tracer tool. The nuclear area was defined manually and the centrosomes were detected automatically by the tracer tool; filament tracer tool determined filament area, filaments length, and branching points per cell. Additionally, a visual assessment was performed to determine the organized status of the spindle actin (aster organization at the highest signal level) and the number of guidance events (spindle actin growth preceding kinetochore fibers growth) was also scored per cell.

### Protein Purification.

Proteins for in vitro assays were purified from HEK293 cells transiently transfected with plasmids expressing EGFP or individual EGFP-FAM110A variants. Two days posttransfection, cells were enriched in mitosis with nocodazole for 12 h, collected by mitotic shake-off and washed with ice-cold PBS. Total cell extracts were obtained by sonicating the pellets in high salt IP buffer (20 mM HEPES pH 7.5, 10 % glycerol, 1 M NaCl, 0.5 % NP40) supplemented with cOmplete protease and PhosSTOP phosphatase inhibitors (Sigma). Cell extracts were then incubated for 3 h at 4 °C with GFP-Trap beads (Chromotek) and washed three times with high salt IP buffer and once with PBS. Bound proteins were eluted from beads by acidic elution with Elution buffer (200 mM Glycine pH 2.4), supernatant was recovered and immediately supplemented with the Neutralization buffer (1 M Tris pH 10.4); protein concentration was measured using Nanodrop and equimolar concentrations were used in all assays.

### Microtubules and F-actin for In Vitro Assays.

Microtubules were prepared as described previously (38). The tubulin ratio used for microtubule preparation was 20 % HiLyte 647 labeled tubulin (TL670M, Cytoskeleton Inc.) and 2 % biotinylated tubulin (T333P, Cytoskeleton Inc.). Unlabeled tubulin was isolated from porcine brains as described previously (39). F-actin was polymerized from unlabeled actin (AKL99, Cytoskeleton Inc.) in polymerization buffer (5 mM HCl-Tris pH 8.0, 0.2 mM CaCl2, 50 mM KCl, 2 mM MgCl2, 1 mM ATP) supplemented with 5 μM mix of labeled and unlabeled phalloidin (R415, P3457, Invitrogen) with ratio of 1:4. Polymerization was done overnight at 4 °C.

### FAM110A Binding to Microtubules and MT-Actin Crosslinking Assay.

Assay channels were constructed using two hydrophobized corning coverslip slides separated by stripes of parafilm as described previously (40). The coverslip surface was functionalized with solution of anti-biotin antibodies (B3640, Sigma, 1 mg/mL in PBS) for 10 min and then immediately passivated for at least 1 h with 10 mg/mL Pluronic F127 solution (Sigma) in PBS. Protein concentration was determined directly in the channel by measuring the GFP signal intensity in solution. Before the experiment, the channel was flushed with 20 µL of assay buffer (50 mM HEPES pH 7.2 KOH, 100 mM KCl, 2 mM MgCl_2_, 1 mM EGTA, 1 mM ATP, 10 mM DTT, 20 mM D-glucose, 0.05 % BSA, 0.1 % Tween20, 0.22 mg/mL glucose oxidase, 0.02 mg/mL catalase), followed by 5 to 10 µL of microtubule solution. The channel was incubated for 5 to 10 s and flushed with 20 µL of assay buffer to remove unbound microtubules. The channel was then flushed with 20 µL protein solution and imaged for 10 min. The same channel was finally used for crosslinking assessment by flushing 20 µL mix of the same protein and labeled actin filaments and was imaged for 10 min.

### F-Actin Bundling Assay.

Assay channels were prepared as described above. Before experiment, channel was flushed with 20 µL of assay buffer followed by a solution of F-actin. Dilution of F-actin filaments was adjusted to achieve single filament coverage on the surface of the coverslip. The channel was incubated for 10 to 20 s and flushed with 20 µL of assay buffer. The channel was then flushed with 20 µL protein solution and imaged for 10 min. For the F-actin network bundling assay, passivated channel without antibody functionalization was flushed with 20 µL of assay buffer followed by F-actin solution in assay buffer supplemented with 0.2 % methylcellulose to keep F-actin in proximity to the coverslip surface. Actin dilution was adjusted to reach an optimal density. Finally, 20 µL of purified EGFP-FAM110A variants in assay buffer was flushed in the channel and imaged for 10 min. The ratio of the F-actin network area was calculated from the last time point value for plotting.

### Imaging of In Vitro Assays and Image Analysis.

In vitro reconstitutions were visualized and performed on Nikon Eclipse Ti2 equipped with an oil immersion Nikon Apo TIRF 60x 1.49 N.A. objective. Fluorophore excitation was achieved with laser wavelengths 640, 561, or 488 nm. Emission filters EM700/75, EM610/75 respectively EM500-545 were used. Images were acquired using either the sCMOS PRIME BSI camera (Teledyne Photometrics) or CMOS Hamamatsu ORCA-flash4.0 LT camera (Hamamatsu Photonics). The binding properties of proteins were quantified as background-subtracted GFP signal density along individual actin filaments. The signal was then normalized to the wild-type FAM110A. Microtubule-actin crosslinking was analyzed as background subtracted signal density of labeled F-actin filaments along microtubules. To measure the normalized actin network area, images were processed as follows: the background was subtracted via the rolling ball method with diameter 50px, stacks were smoothed with gaussian blur with diameter 3px, Otsu threshold was applied to the stack and finally stack was skeletonized. Network area was then normalized to the first frame. To measure normalized actin signal in the bundles, skeletonized network was dilated by 6px and applied as a mask to the original image. Signal was then divided by network area and normalized to the first frame of acquisition.

### F-Actin Quantification in Mitotic Exit.

RPE parental cells or cells stably expressing the EGFP-FAM110A-WT and EGFP-FAM110A-Δ40-61 mutant that were depleted of FAM110A or CK1δ through siRNA and their respective control were arrested in prometaphase using STLC (5 µM) for 16 h and then forced out of mitosis with RO-3306 (20 µM) (8). Alternatively, cells were also treated with CK1 inhibitor PF670462 (1 µM, MedChemExpress) and its respective control. Cells were fixed after 15 min and probed with phalloidin-568, γ-tubulin-647 and DAPI. For quantification, a circular (6 µM diameter) ROI was centered on the centrosomes determined by γ-tubulin staining inside of the chromatin ring determined by DAPI. A total of 17 Z-stack slices with intervals of 0.16 µm were taken per cell, using the centrosome as the middle slice. Mean intensity per slice was measured and the average for each cell. Each dot represents the average phalloidin intensity per cell. All image processing and quantification were performed in ImageJ; statistical significance and plots were done in GraphPad Prism 7.

### Statistics.

Statistical analysis was performed using GraphPad Prism 7. Data are presented as mean ± SD. Statistical significance was evaluated with one-way ANOVA for multiple comparisons, with unpaired two-tailed Student’s *t* tests for comparison of two groups and with one-sample Student’s *t* test for normalized data. Data that did not pass the D’Agostino and Pearson omnibus normality test were compared by the Wilcoxon test (paired) or Mann–Whitney U test. Statistical significance is indicated as *P < 0.05, **P < 0.01, ***P < 0.001, and ****P < 0.0001. All experiments were performed independently at least three times.

## Supplementary Material

Appendix 01 (PDF)

Movie S1. RPE-sAC cells treated with control siRNA (left) and FAM110A siRNA (right); only GFP channel is shown. Time frame intervals are 25 seconds. Scale bars indicate 10 μm.

Movie S2. RPE-sAC cells treated with control siRNA showing a spindle microtubule guidance event by spindle actin. Left panel shows microtubules labeled with SiRTub, middle panel shows spindle actin labeled with sAC-GFP and left panel shows merged microtubules (magenta) and spindle actin (green) channels. Time frame intervals are 25 seconds. Scale bars indicate 10 μm.

Movie S3. RPE-sAC cells treated with FAM110A siRNA showing a spindle microtubule guidance event by spindle actin. Last panel (Merge) shows microtubules (siRTub) in magenta and spindle actin (sAC-GFP) in green. Scale bars indicate 10 μm.

Movie S4. RPE-sAC-GFP-mCherry-FAM110A-WT cells treated with FAM110A siRNA showing the rescue of a spindle microtubule guidance event by spindle actin. Last panel (Merge) shows microtubules (siRTub) in magenta and spindle actin (sAC-GFP) in green. Time frame intervals are 25 seconds. Scale bars indicate 10 μm.

Movie S5. RPE-sAC-GFP-mCherry-FAM110A-Δ40-61 cells treated with FAM110A siRNA showing the failed rescue of proper spindle microtubule guidance event by spindle actin. Lack of spindle actin organization can be appreciated. Last panel (Merge) shows microtubules (siRTub) in magenta and spindle actin (sACGFP) in green. Time frame intervals are 25 seconds. Scale bars indicate 10 μm.

Movie S6. RPE-sAC cells treated with Control siRNA (left) and CLASP2 siRNA (right); only GFP channel for sAC-GFP is shown. Time frame intervals are 25 seconds. Scale bars indicate 10 μm.

Movie S7. RPE-sAC cells treated with Control (left) and PF670462 (right); only GFP channel for sAC-GFP is shown. Time frame intervals are 25 seconds. Scale bars indicate 10 μm.

Movie S8. RPE-sAC control cells showing a spindle microtubule guidance event by spindle actin. Last panel (Merge) shows microtubules (siRTub) in magenta and spindle actin (sAC-GFP) in green. Time frame intervals are 25 seconds. Scale bars indicate 10 μm.

Movie S9. RPE-sAC cells treated with PF670462 inhibitor showing a spindle microtubule guidance event by spindle actin. Last panel (Merge) shows microtubules (siRTub) in magenta and spindle actin (sAC-GFP) in green. Time frame intervals are 25 seconds. Scale bars indicate 10 μm.

## Data Availability

All study data are included in the article and/or supporting information. Raw data were uploaded to BioStudies repository (https://www.ebi.ac.uk/biostudies/) with accession number S-BSST1461 (41).
