# Supplementary material for: FAM110A promotes mitotic spindle formation by linking microtubules with actin cytoskeleton
Source: Proc Natl Acad Sci U S A. 2024 Jul 12;121(29):e2321647121. doi: 10.1073/pnas.2321647121 (PMC11260166; doi:10.1073/pnas.2321647121)
Supplement: Supplementary file 1 — Appendix 01 (PDF) [file pnas.2321647121.sapp.pdf]

FAM110A promotes mitotic spindle formation by linking microtubules with actin cytoskeleton

Cecilia Aquino Perez[1], Mahira Safaralizade[2], Roman Podhajecky[3], Hong Wang[2,4], Zdenek Lansky[3], Robert Grosse[2,4], Libor Macurek[1]

[1] *Cancer Cell Biology, Institute of Molecular Genetics of the Czech Academy of Sciences, Prague CZ14220, Czech Republic*

[2] *Institute for Clinical ad Experimental Pharmacology and Toxicology I, Medical Faculty, University of Freiburg, 79104 Freiburg, Germany*

[3] *Institute of Biotechnology, Czech Academy of Sciences, BIOCEV, Vestec, Czech Republic*

[4] *Centre for Integrative Biological Signaling Studies-CIBSS, University of Freiburg, 79104 Freiburg, Germany*

This PDF file includes

Supplementary Materials and Methods

Supplementary Fig. 1.

Supplementary Fig. 2.

Supplementary Fig. 3.

Supplementary Fig. 4.

Legend to Supplementary movie S1.

Legend to Supplementary movie S2.

Legend to Supplementary movie S3.

Legend to Supplementary movie S4.

Legend to Supplementary movie S5.

Legend to Supplementary movie S6.

Legend to Supplementary movie S7.

Legend to Supplementary movie S9.

Other Supplementary files include

Supplementary movies S1, S2, S3, S4, S5, S6, S7, S8, S9

Supplementary Materials and Methods

Antibodies. The following antibodies were used: FAM110A (mouse monoclonal, clones F4 and B11, Santa Cruz Biotechnology), FAM110A (rabbit polyclonal, Novus Biotechnology), γ-tubulin (rabbit polyclonal, Sigma Aldrich), γ-tubulin (clone GTU-88, Sigma Aldrich), α-tubulin (mouse monoclonal Biorbyt), α-tubulin (clone DM1A, Santa Cruz), and β-actin (clone D6A8, Cell Signaling).

Plasmids. pEGFP-FAM110A plasmid for expression of mouse FAM110A has been described previously (1). The pEGFP-FAM110A-Δ40-61 mutant carrying the deletion of sequence coding aa40-61 and the pEGFP-FAM110A-Δ188-221 mutant carrying the deletion of sequence coding aa188-221 were generated by Gibson assembly. Alternatively, mCherry sequence was cloned in frame into the wild type or mutant FAM110A plasmids and the coding sequence was subcloned into pSBtet-Pur plasmid (Addgene ID: 60507).

DNA sequence alignment. Conservation scoring was performed using the multiple sequence alignment software PRALINE (https://www.ibi.vu.nl/programs/pralinewww/) using the FAM110A orthologue protein sequences of 5 chordate organisms. Second alignment was performed using the human FAM110 homologues. The scoring scheme ranges from 0 (deep blue) for the least conserved alignment position up to 10 (red) for the most conserved.

Immunofluorescence microscopy. Immunofluorescence microscopy was performed as described previously (1, 2). Briefly, RPE parental cells and the EGFP-FAM110A and mCherry-FAM110A variants stable cells grown on glass coverslips were fixed with 4 % paraformaldehyde for 15 min at RT, washed with PBS and permeabilized with 0.15 % Triton X-100 for 15 min. Alternatively, cells were fixed with ice cold methanol for 5 min. Blocking was performed by 3 % BSA in PBS for 30 min. Coverslips were incubated with primary antibodies for 3 h at RT or overnight at 4°C, washed with PBS and incubated with Alexa Fluor-conjugated antibodies for 1 h at RT. After washing with PB, coverslips were incubated with conjugated phalloidin (1 µg/ml) for 20 min, and/or with DAPI for 10 min at RT and mounted using Vectashield. Imaging was performed in a Leica SP8 confocal microscope equipped with HC PL APO 63x/1.40 oil objective. Prometaphase and metaphase cells were imaged using 5 X zoom and 0.16 µm z-stacks. Images were analyzed and processed by LAS AF Lite and ImageJ/Fiji software.

Quantification of FAM110A localization. Cells stably expressing EGFP-FAM110A-WT, -Δ41-61, or -Δ188-221 mutants were treated with MG132 for 30 min prior fixation, stained with γ-Tubulin antibody and Phalloidin-568 and were imaged by confocal microscopy. Mean pixel intensity of EGFP was measured using the spherical region of interest generated by masking γ-Tubulin signal. Similarly, for measurement

of EGFP enrichment of FAM110A-variants at the cell cortex, mean pixel intensity of EGFP was measured using the region of interest generated by masking phalloidin-568 signal. In both cases z-stacks were taken with intervals of 0.16 µm to retrieve the complete structures. Mean intensity per slice was measured and the average for each cell was obtained as the final data point.

Microscopy of mitotic spindles. Cells stably expressing the EGFP-FAM110A-WT, -Δ41-61, or -Δ188-221 mutants were enriched in metaphase prior fixation, stained for α-Tubulin and imaged by confocal microscopy. Intensity density of α-Tubulin-568 was measured by defining a circular (15 µm diameter) ROI that was centered to contain the complete mitotic spindle structure. Z-stack slices were imaged with intervals of 0.16 µm and used to measure the mean intensity density of the complete mitotic spindle structure. For the pole-to-pole distance measurement, fixed cells that were arrested in metaphase were stained with γ-Tubulin and imaged by confocal microscopy; distance between poles was calculated as the hypotenuse (c) from the linear distance between the spindle poles (b) and the relative height distance of both spindle poles (a) as previously described (3).

Flow cytometry (FACS). Determination of the mitotic fraction of cells was performed as described (1). Briefly, **c**ells were fixed with 70 % ice-cold EtOH, permeabilized by 0.5 % Triton X-100 for 15 min at RT, and blocked with 1 % BSA for 30 min. Subsequently, cells were incubated with pMPM-Cy5-conjugated antibody (Cell Signaling, 1:500) for 1 h at RT, washed with PBS, and re-suspended in PBS supplemented with DAPI (5 µg/ml). FACS analysis was performed using LSR II (BD Biosciences) and the FlowJo 10 software (Tree Star, BD). Single cells were gated using SSC and FSC, DAPI staining was used to measure DNA content and to differentiate between G1 and G2 phase cells and mitotic cells were quantified using Cy5 channel.

Immunoprecipitation. HEK293 cells were transiently transfected with plasmids expressing EGFP or individual EGFP-FAM110A or EGFP-FAM110B variants. Two days post transfection, cells were enriched in mitosis with nocodazole for 12 h. Cells were collected by mitotic shake-off and mitotic pellet was washed with ice-cold PBS. Total cell extracts were obtained by sonicating the pellets in CO- IP buffer (20 mM HEPES pH 7.5, 10 % glycerol, 150 mM NaCl, 0.5% NP40) supplemented with cOmplete protease and PhosSTOP phosphatase inhibitors (Sigma). Cell extracts were then incubated for 3 h at 4°C with GFP-Trap beads (Chromotek), washed three times with CO-IP buffer and once with ice cold PBS. For CO-IP assays beads were boiled in sample buffer and analyzed through immunoblotting. For immunoprecipitation with supplemented soluble Actin and Tubulin assay, beads were washed 3 times with high salt (1M NaCl) IP buffer and once with ice cold PBS. Consequently they were provided with supplemented CO-IP buffer plus

1 µM soluble actin or 1 µM soluble Tubulin and incubated for 3 h at 4°C. After 3 washed with simple CO-IP buffer, beads-bound proteins were boiled in sample buffer and analyzed through immunoblotting.

1. C. Aquino Perez, M. Burocziova, G. Jenikova, L. Macurek, CK1-mediated phosphorylation of FAM110A promotes its interaction with mitotic spindle and controls chromosomal alignment. *EMBO reports* **22**, e51847 (2021).
2. C. Aquino Perez, M. Palek, L. Stolarova, P. von Morgen, L. Macurek, Phosphorylation of PLK3 Is Controlled by Protein Phosphatase 6. *Cells* **9** (2020).
3. F. Toyoshima, E. Nishida, Integrin-mediated adhesion orients the spindle parallel to the substratum in an EB1- and myosin X-dependent manner. *Embo j* **26**, 1487-1498 (2007).

Suppl. Fig. 1

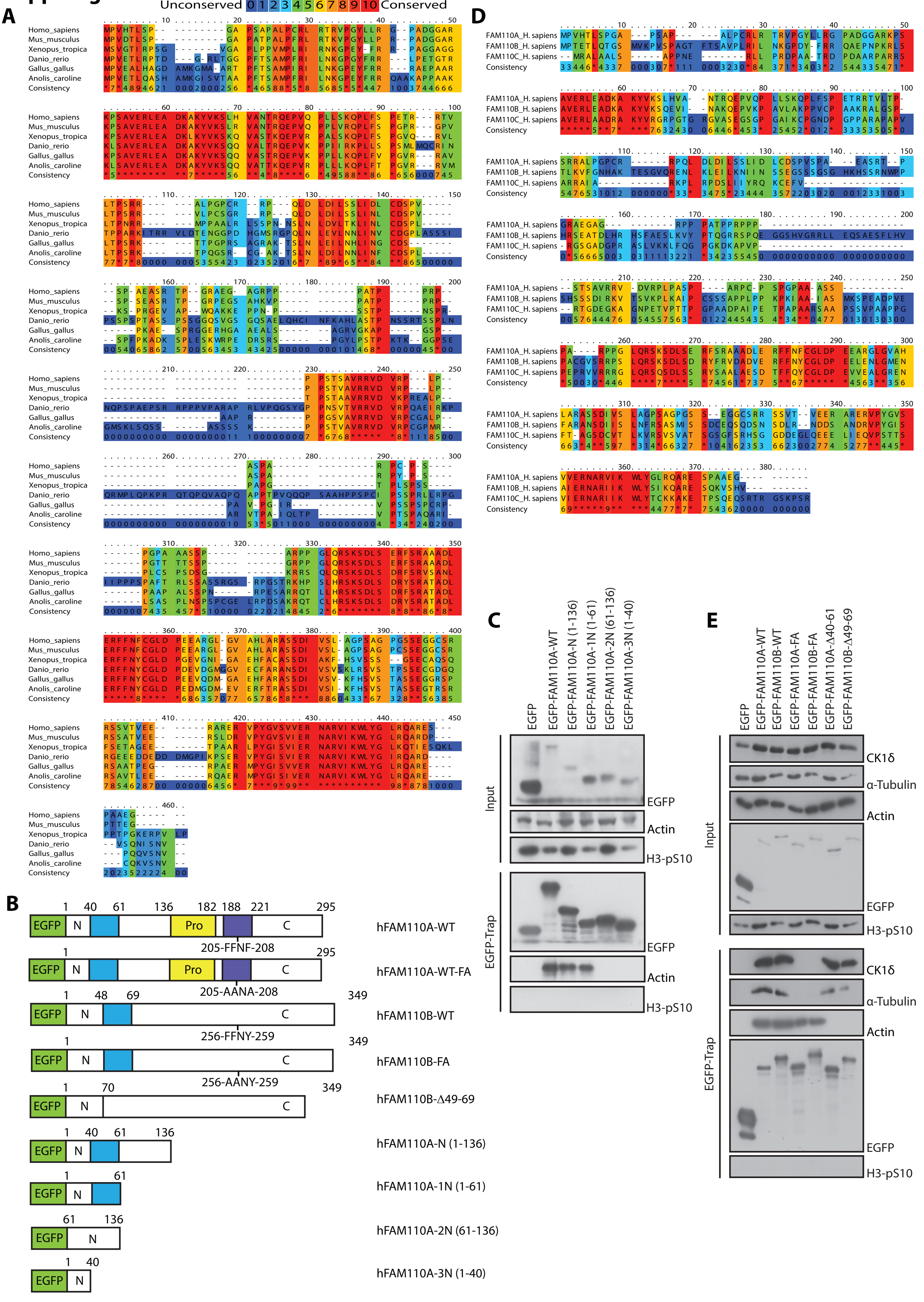

A
Unconserved 0 1 2 3 4 5 6 7 8 9 10 Conserved
Homo_sapiens
Mus_musculus
Xenopus_tropica
Danio_rerio
Gallus_gallus
Anolis_caroline
Consistency
B
hFAM110A-WT
hFAM110A-WT-FA
hFAM110B-WT
hFAM110B-FA
hFAM110B-Δ49-69
hFAM110A-N (1-136)
hFAM110A-1N (1-61)
hFAM110A-2N (61-136)
hFAM110A-3N (1-40)
EGFP
N
C
Pro
205-FFNF-208
205-AANA-208
256-FFNY-259
256-AANY-259
C
EGFP
EGFP-FAM110A-WT
EGFP-FAM110A-N (1-136)
EGFP-FAM110A-1N (1-61)
EGFP-FAM110A-2N (61-136)
EGFP-FAM110A-3N (1-40)
Input
EGFP-Trap
Actin
H3-pS10
D
FAM110A_H. sapiens
FAM110B_H. sapiens
FAM110C_H. sapiens
Consistency
E
EGFP-FAM110B-WT
EGFP-FAM110A-FA
EGFP-FAM110B-FA
EGFP-FAM110A-Δ40-61
EGFP-FAM110B-Δ49-69
CK1δ
α-Tubulin

Supplementary Fig. 1.

A. Family with sequence similarity 110 member A (FAM110A) orthologues sequence alignment from human and of 5 model organism representing each common vertebrate classes. FAM110A presents four highly conserved regions, two in the N-terminal domain (Ser41 to Glu-65 and Leu102 - to Asn112) and two in the C-terminal domain (Ser-188 to Gly-221 and Pro-266 to Gly-284). The scoring scheme works from 0 (deep blue) for the least conserved alignment position up to 10 (red) for the most conserved.

B. Scheme of the EGFP-FAM110A constructs used to map down and confirm FAM110A interaction with actin. Numbering is based on human FAM110A. N, C, and Pro represent N-terminal, C-terminal, and Pro-rich domains, respectively. Blue area represents conserved α-helix Ser41 to Glu-65 and purple area represents conserved α-helix Ser-188 to Gly-221.

C. Representative immunoblot of co-IP assay performed in transiently transfected HEK293 with EGFP, EGFP-FAM110A-WT and EGFP-FAM110A N-terminal domain truncations (*n* = 3). Antibody against pS10-H3 was used as a marker of mitosis.

D. Human Family with sequence similarity 110 (FAM110) homologues alignment.

E. Representative immunoblot of co-IP assay performed in transiently transfected HEK293 with EGFP as control, EGFP-FAM110A-WT, EGFP-FAM110A-Δ40-61, EGFP-FAM110A-FA, EGFP-FAM110B-WT, EGFP-FAM110B-Δ49-69 and EGFP-FAM110B-FA (*n* = 3). Antibody against pS10-H3 was used as a marker of mitosis.

Suppl. Fig. 2

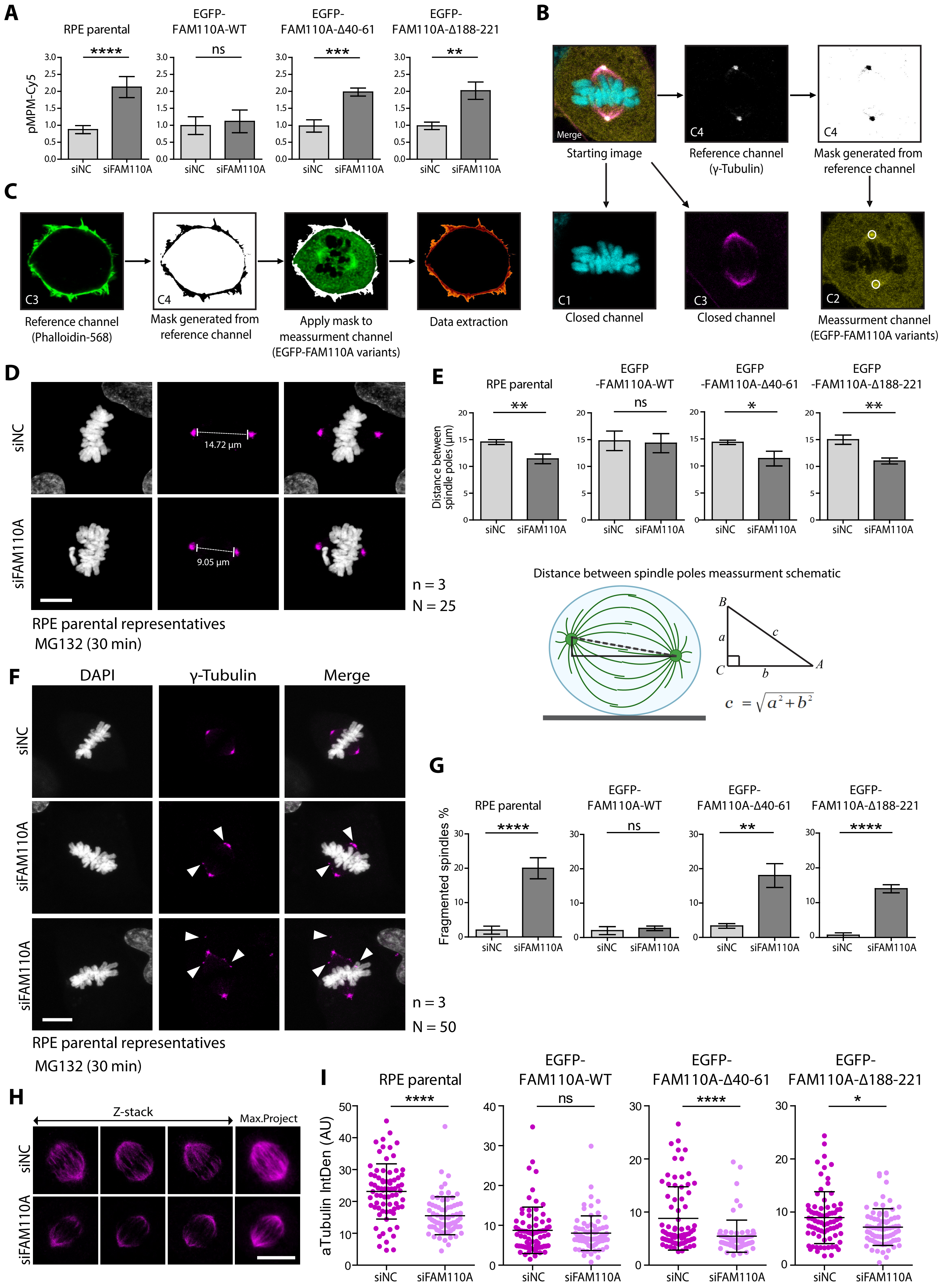
A
RPE parental
EGFP-FAM110A-WT
EGFP-FAM110A-Δ40-61
EGFP-FAM110A-Δ188-221
pMPM-Cy5
****
ns
***
**
siNC
siFAM110A
B
Merge
Starting image
C4
Reference channel (γ-Tubulin)
C4
Mask generated from reference channel
C1
Closed channel
C3
Closed channel
C2
Meassurment channel (EGFP-FAM110A variants)
C
C3
Reference channel (Phalloidin-568)
C4
Mask generated from reference channel
Apply mask to meassurment channel (EGFP-FAM110A variants)
Data extraction
D
siNC
siFAM110A
14.72 µm
9.05 µm
n = 3
N = 25
RPE parental representatives
MG132 (30 min)
E
RPE parental
EGFP -FAM110A-WT
EGFP -FAM110A-Δ40-61
EGFP -FAM110A-Δ188-221
Distance between spindle poles (µm)
**
ns
*
**
siNC
siFAM110A
Distance between spindle poles meassurment schematic
B
a
c
C
b
A
$c = \sqrt{a^2+b^2}$
F
DAPI
γ-Tubulin
Merge
siNC
siFAM110A
siFAM110A
n = 3
N = 50
RPE parental representatives
MG132 (30 min)
G
RPE parental
EGFP-FAM110A-WT
EGFP-FAM110A-Δ40-61
EGFP-FAM110A-Δ188-221
Fragmented spindles %
****
ns
**
****
siNC
siFAM110A
H
Z-stack
Max.Project
siNC
siFAM110A
I
RPE parental
EGFP-FAM110A-WT
EGFP-FAM110A-Δ40-61
EGFP-FAM110A-Δ188-221
aTubulin IntDen (AU)
****
ns
****
*
siNC
siFAM110A

Supplementary Fig. 2.

A. RPE parental cells and cells stably expressing the EGFP-FAM110A-WT, EGFP-FAM110A-Δ40-61 and EGFP-FAM110A-Δ188-221 mutant were transfected with control (siNC) or FAM110A siRNA. After 48 h, fraction of mitotic cells was determined by flow cytometry using staining for pS10-H3 and DAPI. Shown is median ± SD. Statistical significance was determined by two-tailed *t*-test ($n = 3$, $^{**}P < 0.005$).

B. Schematic representation of how EGFP spindle enrichment was observed, quantified and analyzed using ImageJ/Fiji. γ-tubulin channel was used to create a mask to delimitate the volume were EGFP signal was measured.

C. Schematic representation of how EGFP cell cortex enrichment was observed, quantified and analyzed using ImageJ/Fiji. Phalloidin channel was used to create a mask to delimitate the volume were EGFP signal was measured.

D. Representative RPE parental cells from (E). Cells transfected with control or FAM110A siRNA were fixed with 4% PFA after 48 h, and stained for γ-tubulin-647 and DAPI. Middle panel shows a representative measurement between poles. Metaphase cells were imaged by confocal microscopy, scale bar indicates 10 µm.

E. RPE parental cells and cells stably expressing the EGFP-FAM110A-WT, EGFP-FAM110A-Δ40-61 and EGFP-FAM110A-Δ188-221 mutants were used to quantify the linear distance between mitotic spindle poles during metaphase. Poles were stained with γ-tubulin-647 and manually measured using the scale tool in ImageJ/Fiji (N=25). Shown is median ± SD ($n = 3$). Statistical significance was determined by two-tailed *t*-test ($^{**}P < 0.01$). $^{*}P < 0.05$).

F. Representative RPE parental cells from (G). Cells transfected with control or FAM110A siRNA were fixed with 4% PFA after 48 h, and stained for γ-tubulin-647 and DAPI. White arrowheads show abnormal fragmented spindles. Metaphase cells were imaged by confocal microscopy, scale bar indicates 10 µm.

G. RPE parental cells and cells stably expressing the EGFP-FAM110A-WT, EGFP-FAM110A-Δ40-61 and EGFP-FAM110A-Δ188-221 mutant were transfected with control (siNC) or FAM110A siRNA. After 48 cells were treated with MG132 for 30 min prior fixation. Fragmentation of spindle poles was scored in 50 metaphase cells per condition. Error bars indicate median ± SD. Statistical significance was determined by one-way ANOVA ($n = 3$, $^{****}P < 0.0001$ and $^{**}P < 0.01$).

H. Representative RPE parental cells from (G). Cells transfected with control or FAM110A siRNA were fixed with 4% PFA after 48 h, and stained for α-tubulin-568 and DAPI. First three panels show Z-stack

range; last panel at the right shows the maximum projection. Metaphase cells were imaged by confocal microscopy, scale bar indicates 10 µm.

I. RPE parental cells and cells stably expressing the EGFP-FAM110A-WT, EGFP-FAM110A-Δ40-61 and EGFP-FAM110A-Δ188-221 mutants were transfected with control (siNC) or FAM110A siRNA and stained with α-tubulin-568. Mitotic spindle density was quantified in metaphase cell. Each dot represents an individual mitotic spindle (N=25), shown is median ± SD (n = 3). Statistical significance was determined by one-sample t-test (*$P < 0.05$).

Suppl. Fig. 3

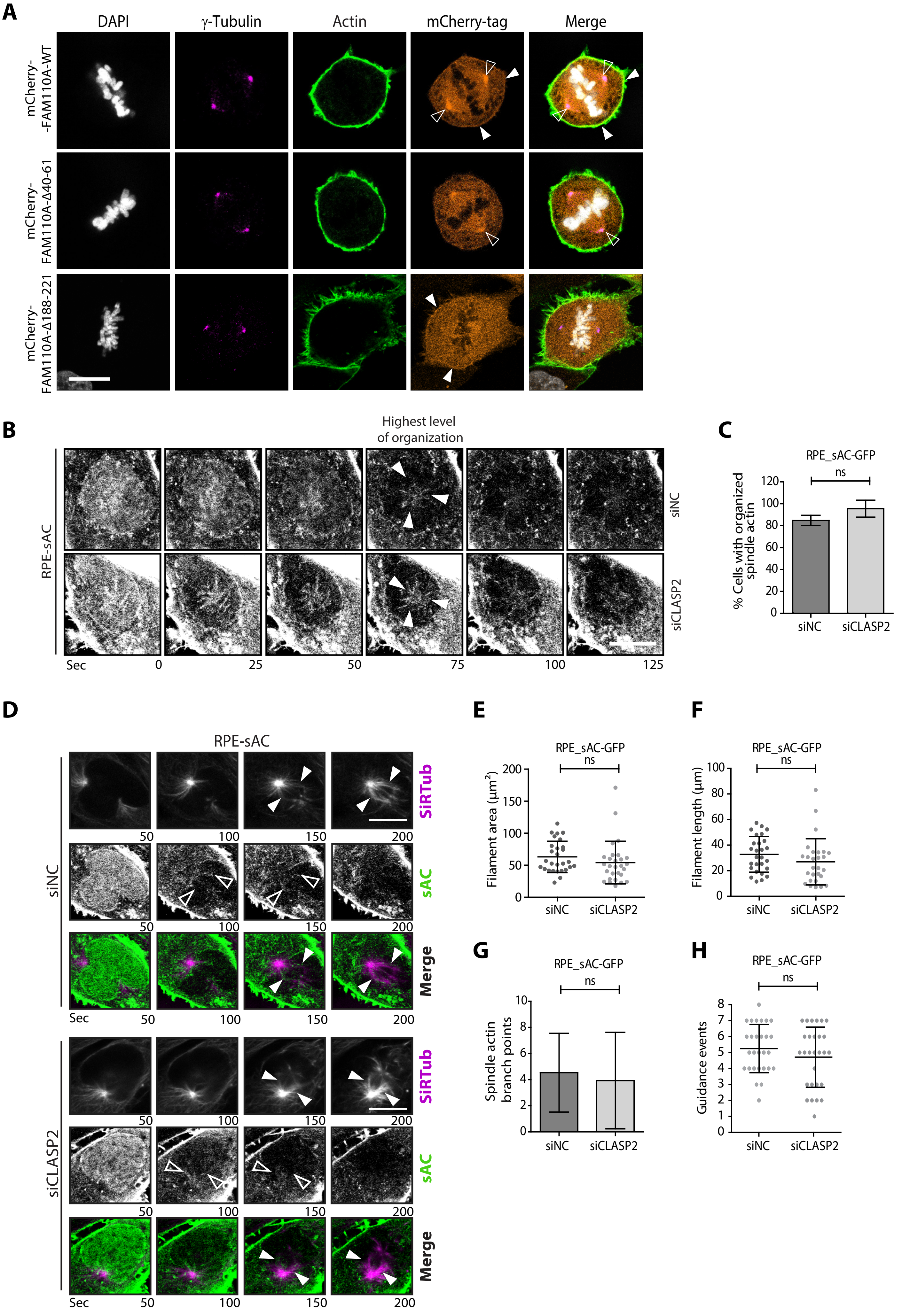
A
DAPI
γ-Tubulin
Actin
mCherry-tag
Merge
mCherry--FAM110A-WT
mCherry-FAM110A-Δ40-61
mCherry-FAM110A-Δ188-221
B
Highest level of organization
RPE-sAC
siNC
siCLASP2
Sec 0 25 50 75 100 125
C
RPE_sAC-GFP
ns
% Cells with organized spindle actin
siNC
siCLASP2
D
RPE-sAC
siNC
siCLASP2
SiRTub
sAC
Merge
Sec 50 100 150 200
E
RPE_sAC-GFP
ns
Filament area (μm²)
siNC
siCLASP2
F
RPE_sAC-GFP
ns
Filament length (μm)
siNC
siCLASP2
G
RPE_sAC-GFP
ns
Spindle actin branch points
siNC
siCLASP2
H
RPE_sAC-GFP
ns
Guidance events
siNC
siCLASP2

Supplementary Fig. 3.

A. Cells stably expressing the mCherry-FAM110A-WT, mCherry-FAM110A-Δ40-61 and mCherry-FAM110A-Δ188-221 mutants were fixed and stained for DAPI, γ-tubulin-647 and actin with phalloidin-568 and analyzed by confocal microscopy. Representative images of a single stack of metaphase cells are shown. Full arrowheads show colocalization with cell cortex actin and empty arrowhead show enrichment at spindle poles. Scale bar indicate 10 µm.

B. Representative live image sequences of RPE-sAC cells transfected with control (siNC) or CLASP2 siRNA. Shown is GFP channel and numbers show time in seconds, time 0 was set to indicate NEB at the onset of prophase. Arrowheads indicate spindle actin with the highest level of organization. Scale bar indicate 10 µm.

C. Quantification of spindle actin formation in B and D. Plotted is the percentage of cells that presented an organized actin spindle for each independent repetition ± SD. Statistical significance was determined by t-test (*n=3*).

D. Representative live image sequences of RPE-sAC cells transfected with control (siNC) or CLASP2 siRNA and MT stained by SiR-Tub. Arrowhead shows a kinetochore MT fiber (magenta), empty arrowhead shows event where spindle actin (green) preceded formation of kinetochore MT. Scale bars indicate 10 µm.

E. Quantification of the spindle actin area in B and D using Imaris software - filament tracer tool. Plotted is the total area covered by the organized spindle actin structure (μm^2). Bars indicate mean ± SD, each dot represents a single cell. Statistical significance was determined by t-test.

F. Quantification of the F-actin length in B and D using Imaris software - filament tracer tool. Plotted is the total sum of the length of all detected actin filaments (µm) per cell ± SD, each dot represents a single cell. Statistical significance was determined by t-test.

G. Quantification of spindle actin branch points in B, and D using Imaris software. Plotted is the mean of the observed actin branch points per cell ± SD. Statistical significance was determined by t-test.

H. Quantification of actin-MT guidance events in B and D. Plotted is the number of events when spindle actin preceded growth of a kinetochore MT per cell. Bars indicate mean ± SD, each dot represents a single cell. Statistical significance was determined by t-test.

Suppl. Fig. 4

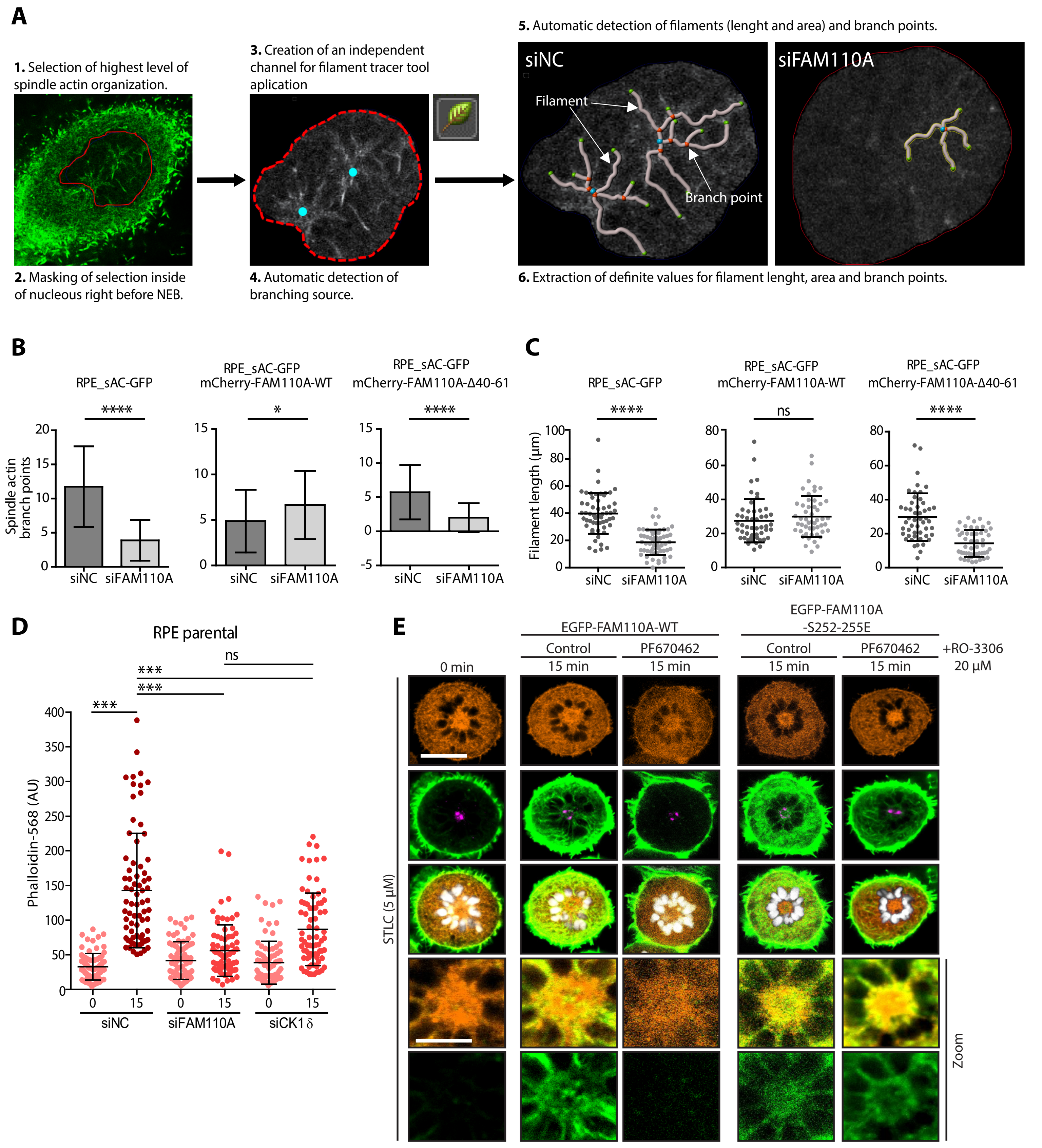

A
1. Selection of highest level of spindle actin organization.
2. Masking of selection inside of nucleous right before NEB.
3. Creation of an independent channel for filament tracer tool aplication
4. Automatic detection of branching source.
5. Automatic detection of filaments (lenght and area) and branch points.
siNC
siFAM110A
Filament
Branch point
6. Extraction of definite values for filament lenght, area and branch points.
B
RPE_sAC-GFP
RPE_sAC-GFP mCherry-FAM110A-WT
RPE_sAC-GFP mCherry-FAM110A-Δ40-61
Spindle actin branch points
****
*
****
siNC siFAM110A
C
Filament length (μm)
****
ns
****
siNC siFAM110A
D
RPE parental
Phalloidin-568 (AU)
***
ns
0 15 0 15 0 15
siNC siFAM110A siCK1δ
E
EGFP-FAM110A-WT
EGFP-FAM110A -S252-255E
0 min
Control 15 min
PF670462 15 min
+RO-3306 20 μM
STLC (5 μM)
Zoom

Supplementary Fig. 4.

A. Schematic representation of how spindle actin was observed, quantified and analyzed using IMARIS filament tracer tool. In the films obtained from the *in vivo* imaging, the frame showing the highest level of organization was selected (1). The area inside the nucleus was masked and extracted in a separate channel were filaments tracer tool was implemented (2, 3). First the source point of actin branches was automatically identified as blue dots that show localization of the centrosomes (2). Subsequently segments (gray) and branch points (orange) were identified based in local intensity thresholding through background subtraction (5). The definite values for filament length, filament area and branch point per cell were extracted and plotted in GraphPad Prism 7, were statistical analysis was also performed (6).

B. Quantification of the F-actin length in Fig. 4B and D using Imaris software. Plotted is the total sum of the length of all detected actin filaments (µm) per cell ± SD, each dot represents a single cell. Statistical significance was determined by t-test (n = 3, N=25, ****P < 0.0001).

C. Quantification of the F-actin length in Fig. 4B and D using Imaris software - filament tracer tool. Plotted is the total sum of the length of all detected actin filaments (µm) per cell ± SD, each dot represents a single cell. Statistical significance was determined by t-test (n = 3, N=25, ****P < 0.0001).

D. Quantification of actin growth around the centrosomes after forced mitotic exit after FAM110A or CK1δ depletion. Plotted is mean of Phalloidin-568 intensity around the monopolar centrosome at 0 or 15 minutes time point ± SD (each dot represents the mean value of all stacks of each single cell). Statistical significance was determined by one-way ANOVA (n = 3) (N=25) (***P < 0.001).

E. Representative images of F-actin formation during the mitotic exit. EGFP-FAM110A-WT and EGFP-FAM110A-S252-253 stable cell lines were arrested in prometaphase using STLC (5 µM) and were treated with Control or PF6700462 (1 µM). Then cells were forced to exit mitosis by RO-3306 and fixed after 15 min. DNA was stained with DAPI (white), actin with phalloidin (green) and centrosomes were labeled with γ-tubulin (magenta). Scale bars indicate 10 µm for full panels and 5 µm for zoom panels.

VIDEO LEGENDS

MOVIE 1. RPE-sAC cells treated with control siRNA (left) and FAM110A siRNA (right); only GFP channel is shown. Time frame intervals are 25 seconds. Scale bars indicate 10 µm.

MOVIE 2. RPE-sAC cells treated with control siRNA showing a spindle microtubule guidance event by spindle actin. Left panel shows microtubules labeled with SiRTub, middle panel shows spindle actin labeled with sAC-GFP and left panel shows merged microtubules (magenta) and spindle actin (green) channels. Time frame intervals are 25 seconds. Scale bars indicate 10 µm.

MOVIE 3. RPE-sAC cells treated with FAM110A siRNA showing a spindle microtubule guidance event by spindle actin. Last panel (Merge) shows microtubules (siRTub) in magenta and spindle actin (sAC-GFP) in green. Scale bars indicate 10 µm.

MOVIE 4. RPE-sAC-GFP-mCherry-FAM110A-WT cells treated with FAM110A siRNA showing the rescue of a spindle microtubule guidance event by spindle actin. Last panel (Merge) shows microtubules (siRTub) in magenta and spindle actin (sAC-GFP) in green. Time frame intervals are 25 seconds. Scale bars indicate 10 µm.

MOVIE 5. RPE-sAC-GFP-mCherry-FAM110A-Δ40-61 cells treated with FAM110A siRNA showing the failed rescue of proper spindle microtubule guidance event by spindle actin. Lack of spindle actin organization can be appreciated. Last panel (Merge) shows microtubules (siRTub) in magenta and spindle actin (sAC-GFP) in green. Time frame intervals are 25 seconds. Scale bars indicate 10 µm.

MOVIE 6. RPE-sAC cells treated with Control siRNA (left) and CLASP2 siRNA (right); only GFP channel for sAC-GFP is shown. Time frame intervals are 25 seconds. Scale bars indicate 10 µm.

MOVIE 7. RPE-sAC cells treated with Control (left) and PF670462 (right); only GFP channel for sAC-GFP is shown. Time frame intervals are 25 seconds. Scale bars indicate 10 µm.

MOVIE 8. RPE-sAC control cells showing a spindle microtubule guidance event by spindle actin. Last panel (Merge) shows microtubules (siRTub) in magenta and spindle actin (sAC-GFP) in green. Time frame intervals are 25 seconds. Scale bars indicate 10 µm.

MOVIE 9. RPE-sAC cells treated with PF670462 inhibitor showing a spindle microtubule guidance event by spindle actin. Last panel (Merge) shows microtubules (siRTub) in magenta and spindle actin (sAC-GFP) in green. Time frame intervals are 25 seconds. Scale bars indicate 10 µm.
